# Enhancement of wheat resistance to dry–hot wind stress during grain filling by 24-epibrassinolide: optimization of hormone balance and improvement of flag leaf photosynthetic performance

**DOI:** 10.3389/fpls.2025.1552617

**Published:** 2025-02-24

**Authors:** Chao Wang, Haixing Cui, Min Jin, Jiayu Wang, Chunhui Li, Yong Li, YongLi Luo, Zhenlin Wang

**Affiliations:** ^1^ State Key Laboratory of Wheat lmprovement, Shandong Agricultural University, Taian, China; ^2^ College of Agronomy, Shandong Agricultural University, Taian, China

**Keywords:** wheat, 24-epibrassinolide, dry-hot wind, hormone, antioxidant enzyme

## Abstract

**Introduction:**

Dry-hot wind during the grain filling period is a prevalent agrometeorological challenge worldwide, causing significant functional leaf senescence, disrupting the grain filling process, and ultimately leading to wheat yield loss. Although studies have explored the alleviating effects of EBR under abiotic stress, its application in wheat and the potential mechanisms underlying its role in mitigating dry–hot wind still require further investigation.

**Methods:**

Using the dry–hot-wind-sensitive cultivar Jinan 17 (JN17) and the dry–hot-wind-resistant cultivar Liangxing 77 (LX77) as experimental materials. A split-plot design was employed, with cultivar as the main plot factor, dry-hot wind (DH) treatment as the subplot factor, and the foliar application of 24-epibrassinolide (EBR) at a concentration of 0.1 mg L^-1^ as the sub-subplot factor.

**Results:**

EBR alleviated the negative effects of DH stress on wheat by optimizing the hormone balance. The abscisic acid (ABA) and jasmonic acid (JA) contents decreased, and the salicylic acid (SA) content increased, which promoted the stomatal opening of flag leaves. The transpiration rate (Tr) was increased by 7–10% and thus reduced the temperature of the spikes and leaves by 0.87–1.27 °C and 1.6–2.4 °C, respectively. Additionally, the activities of antioxidant enzymes, including superoxide dismutase (SOD), peroxidase (POD), and catalase (CAT), were enhanced, which prevented early flag leaf senescence and maintained a high chlorophyll level under DH stress. Therefore, the photosynthetic performance of flag leaves was enhanced. EBR enhanced the yield of JN17 and LX77 under DH stress. JN17’s yield was increased by 9.2% and 7.5% in the 2020–2021 and 2021–2022 wheat growing seasons, respectively, and LX77’s yield was increased by 4.9% and 2.3% over two years.

**Discussion:**

This study provides a promising approach for enhancing wheat’s resistance to DH stress, with practical implications for wheat production.

## 1 Introduction

Wheat (*Triticum aestivum* L.) is a staple crop in the world, and its yield is intimately tied to local climatic conditions. Elevated temperatures during the grain filling stage elicit heat stress in wheat, severely affecting grain yield (Li et al., 2023). Dry–hot wind (DH), a combination of high-temperature stress, drought stress, and wind force, particularly during the latter stages of wheat growth. This phenomenon damages chloroplasts and protein structures, reduces chlorophyll synthesis, and decreases photosynthetic rates. It also intensifies transpiration, leading to water loss, and disrupts biological membranes, reducing antioxidant enzyme activities. Furthermore, it impedes organic matter accumulation and compromises root vitality, ultimately resulting in a decline in wheat yield (Akter and Rafiqul Islam, 2017). Hence, investigating the effects of DH on wheat and exploring efficacious strategies to bolster wheat’s resilience against DH are imperative for ensuring sustainable wheat production.

Brassinosteroids (BRs), a fundamental class of natural steroid hormones, have been universally recognized as the sixth major class of plant growth regulators, exhibiting a wide range of bioactivities (Peres et al., 2019). They play a central role in orchestrating plant growth and development (Clouse, 1996; Yang et al., 2011; Nolan et al., 2017; Dong et al., 2020), promoting cell elongation, modulating cell division and differentiation, facilitating reproductive organogenesis (Tanveer et al., 2018), regulating photosynthesis, and enhancing plant tolerance to abiotic stresses (Kang et al., 2017; Retzer et al., 2019). Although previous studies have demonstrated the pivotal role of BRs, including 24-epibrassinolide (EBR), in improving crop resilience against abiotic stresses such as drought, heat stress, and salinity (Yang et al., 2021; Zhong et al., 2023; Khan et al., 2024), investigations specifically addressing EBR application under dry–hot wind conditions remain insufficient, with relevant studies being notably scarce.

Plant hormones play an indispensable role in responding to abiotic stress. Previous studies have shown that there are synergistic interactions between BRs and other hormones. For example, BRs and gibberellins (GAs) share similar roles in developmental processes, such as seed germination and apical hook development (Zhao et al., 2021; Xiong et al., 2021, 2022). Under normal conditions, BRs enhance the expression of GA biosynthesis genes, leading to increased GA accumulation (Unterholzner et al., 2015). However, in rice, BRs reduce GA biosynthesis by repressing *GA20ox3*. In maize, jasmonic acid (JA) regulates the bHLH network by attenuating BR signaling to suppress *ZmXTH1* expression, thus regulating cell elongation (Wang et al., 2024). Additionally, BRs and JA interact synergistically in response to virus infection in rice (Hu et al., 2020). Under normal conditions, BRs antagonistically interact with abscisic acid (ABA) (Hussain et al., 2023), and exogenous BL treatment can lower ABA levels by downregulating the transcription of ABA biosynthesis genes (Ha et al., 2018). In Arabidopsis, BRs interact with salicylic acid (SA) to regulate plant immune responses (Kim et al., 2022). At the same time, under abiotic stress conditions, an excessive accumulation of ROS in plants leads to cellular damage (Anjum et al., 2016; Soares et al., 2016; Guo et al., 2017; Czarnocka and Karpiński, 2018; Kaur et al., 2019; Sharma et al., 2019). To counteract this stress and eliminate reactive oxygen species (ROS), plants actively enhance the activities of antioxidant enzymes, such as superoxide dismutase (SOD), peroxidase (POD), and catalase (CAT) (Ruley et al., 2004; Šimonovičová et al., 2004). Research indicates that EBR can significantly enhance the activities of these antioxidant enzymes, effectively reducing cellular damage (Mazorra et al., 2002; Karlidag et al., 2011). Furthermore, BR treatment can decrease the production and accumulation of ROS by regulating the expression levels of related genes, such as by inhibiting the expression of the H_2_O_2_ synthesis gene *RBOH* (Sharma et al., 2017). Thus, EBR not only enhances the activities of antioxidant enzymes but also inhibits the expression of genes related to ROS production, thereby significantly reducing stress-induced damage to plants.

Abiotic stress markedly impacts plant photosynthesis, including reducing chlorophyll synthesis, damaging photosystems I and II and the electron transport chain, decreasing stomatal conductance (gs) and the net photosynthetic rate (Pn), and triggering ROS production, thus inhibiting the activity of ribulose-1,5-bisphosphate carboxylase/oxygenase (Rubisco) (Xia et al., 2006; Chaves et al., 2009; Kohli et al., 2017). BRs play crucial roles in plant responses to abiotic stress by enhancing CO_2_ fixation efficiency and photosystem II (PSII) stability, consequently boosting photosynthesis (Siddiqui et al., 2018). EBR helps in maintaining chloroplast structure and activity while facilitating the uptake of essential ions (such as Ca^2+^ and Mg^2+^) in plants, subsequently promoting chlorophyll synthesis and enhancing photosynthetic efficiency (Choudhary et al., 2012; Alam et al., 2019). Abiotic stress can disrupt plant cell structure, inhibit metabolic processes, weaken photosynthesis, and affect cell membrane and chloroplast functions through stress responses, especially electron transport in thylakoid membranes (Ashraf and Ali, 2008; Lawlor, 2009; Sade et al., 2009). In response to adversity, plants accumulate proline and soluble sugars as carbon and nitrogen sources and osmotic regulators to maintain cell structure and energy supply and to scavenge free radicals (Liu et al., 2009). In soybeans subjected to water stress, BRs analogously enhance water potential through increased soluble sugar and proline contents, ultimately leading to increased biomass accumulation (Zhang et al., 2008). Furthermore, BRs modulate ion balance by decreasing Na^+^ and Cl^-^ contents and increasing K^+^ and Ca^2+^ contents, thereby maintaining osmotic potential and water balance in plants (Liaqat et al., 2020).

In summary, the application of EBR in wheat and its potential mechanisms for alleviating dry hot wind stress require further investigation. Therefore, this experiment simulated dry hot wind stress during the wheat grain-filling stage and studied the responses of the dry-hot-wind-sensitive cultivar JN17 and the dry-hot-wind-resistant cultivar LX77 under EBR treatment. Ultimately, this study aims to elucidate the mechanism by which EBR enhances wheat tolerance to dry–hot wind stress, providing a theoretical basis and technical support for improving wheat resilience under dry–hot wind conditions.

## 2 Materials and methods

### 2.1 Plant growth conditions

The experiment was conducted during the 2020–2021 and 2021–2022 wheat growing seasons at the agronomy experimental farm of Shandong Agricultural University in Tai’an, Shandong Province (36°09′ N, 117°09′ E, elevation 128 m), in a temperate monsoon climate. The soil was classified as Eutriccambisols (WRB, 2015). The preceding crop was corn, and straw incorporation was performed, with an organic matter content of 12.3 g kg^−1^, a total nitrogen content of 0.91 g kg^−1^, an alkali-hydrolyzable nitrogen content of 87.2 mg kg^−1^, an available phosphorus content of 12.6 mg kg^−1^, and an available potassium content of 57.5 mg kg^−1^ in the 0–20 cm soil layer.

### 2.2 Experimental design

The wheat cultivars utilized in the experiment were the dry–hot wind sensitive JN17 and the dry–hot wind resistant variety LX77 (Yi et al., 2015). A split-plot design was employed, with cultivar as the main plot factor, dry-hot wind (DH) treatment as the subplot factor, and the foliar application of 24-epibrassinolide (EBR) at a concentration of 0.1 mg L^−1^ as the sub-subplot factor. The treatments were divided into four treatment combinations: dry–hot wind + spraying water (DH + SW), dry–hot wind + EBR (DH + EBR), field + spraying water (FC + SW), and field + EBR (FC + EBR). Each treatment has three replicates, with a plot area of 9 m² (3 m × 3 m).

DH treatment: In both years, the shelters were constructed in May (with a height of 3.5 m, a width of 7 m, and a length of 22 m, using steel materials). The side walls were made of polymethyl methacrylate (PMMA) panels, and the transmittance could reach 93%. The roof covering of the DH shelters was composed of Po (Polyolefin) film, exhibiting a light transmittance rate exceeding 92%. An automatic temperature and humidity control system was used to create DH conditions inside the shelter, comprising two fan heaters (power: 90 kW; frequency: 50 Hz), two dehumidifiers (power: 1200 W; frequency: 50 Hz), and wall-mounted fans (power: 60 W, frequency: 50 Hz, specification: 400 mm, simulating natural wind, wind speed: 3.5 m s^−1^) installed along the long and short sides at 2.5 m intervals. Two exhaust fans (frequency: 50 Hz; power: 100 W, specification: 415 mm) were installed on the upper sections of the two short walls. When the temperature inside the shelter dropped below 35°C, the fan heaters automatically activated to provide heating, and they stopped when the temperature exceeded 40°C. Subsequently, the side windows and exhaust fans (operating at 1400 rpm) opened to reduce the temperature. In the absence of a cooling requirement, the side windows remained closed, and the exhaust fans operated at 500 rpm to ensure air circulation. The dehumidifiers started when the relative humidity exceeded 30%, and turned off when the humidity fell below 25%. The roof covers and side walls were installed on May 14, followed by the initiation of the dry–hot wind treatment, the treatment was conducted daily from 9:00 a.m. to 4:00 p.m. for 10 consecutive days, after which the shelter was removed to restore natural conditions.

**Figure 1:**
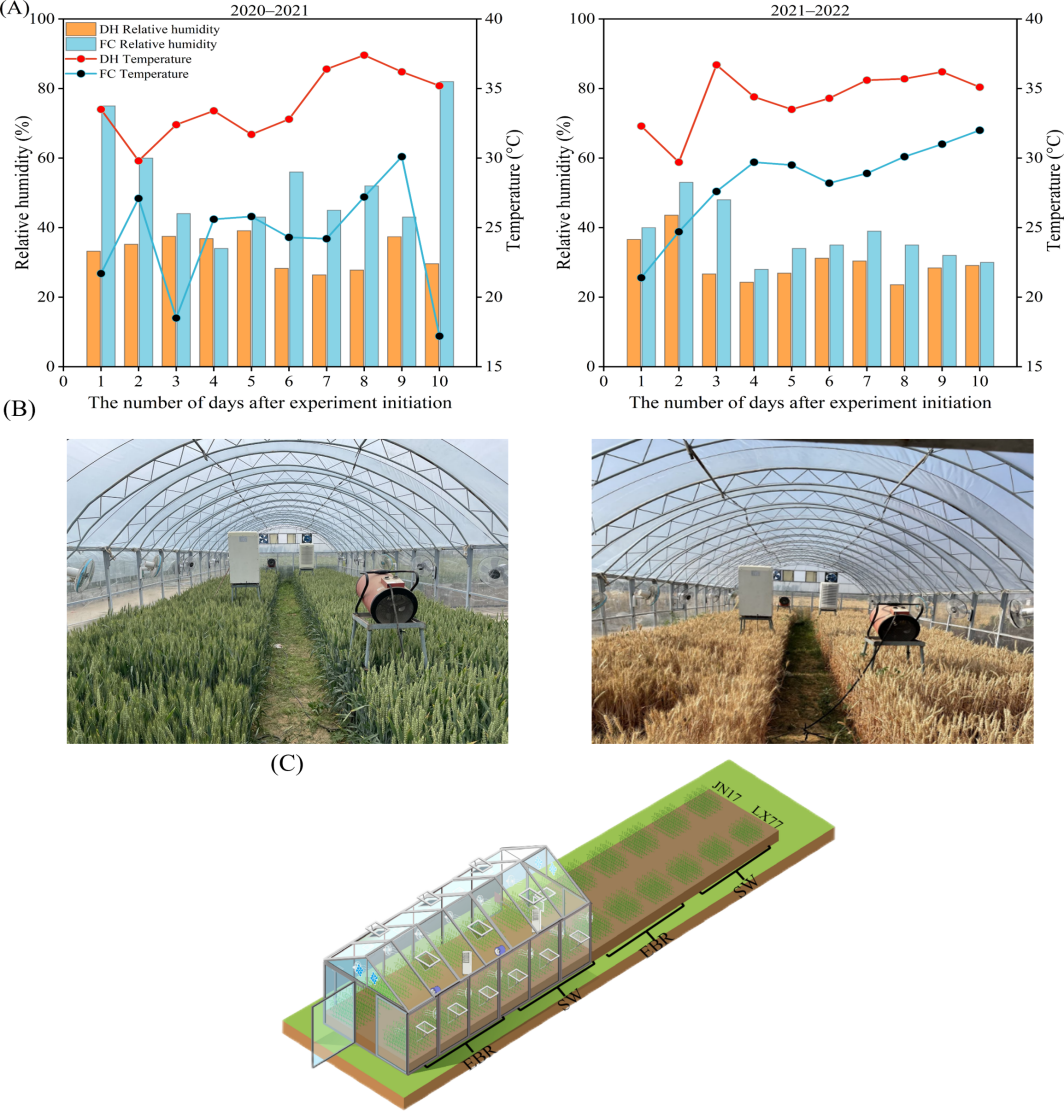
Evolution in temperature and relative humidity inside and outside the dry-hot wind shelter during the wheat growing seasons of 2020–2021 and 2021–2022, along with display of the experimental site. **(A)** Temperature and relative humidity; **(B)** experimental setting; **(C)** sketch of the experimental distribution.

EBR was sprayed according to the following method: the standard product was produced by Solarbio, with a spraying concentration of 0.1 mg L^−1^, and add 1 ml of Tween 20 was added to each liter of solution. The control treatment was SW, with 1 ml of Tween 20 added to each liter of solution. Spraying began on the first day of the DH treatment, with 1 L sprayed continuously per plot, for 3 days during the evening time. **Figures 1A-C** illustrate the temperature and humidity conditions during the DH treatments, a live map of the experimental site, and the layout of the experimental treatments. The precipitation during the DH treatment period was recorded as zero.

In this experiment, manual seeding was employed, and mechanical weeding was conducted before sowing. Phosphorus and the potassium fertilizers superphosphate (P_2_O_5_ 16%) and potassium chloride (K_2_O 60%) were applied at rates of 70 kg ha^−1^ and 120 kg ha^−1^. Urea was used as the nitrogen fertilizer, with a total application rate of 240 kg N ha^−1^; 120 kg N ha^−1^ was applied as a basal fertilizer before sowing, and the remaining 120 kg N ha^−1^was top-dressed at the jointing stage. The phosphorus and potassium fertilizers were applied entirely as base fertilizers, along with the nitrogen base fertilizer. The planting density was 300 plants m^−2^, the basic seedling (the number of seedlings that successfully germinated and became established within a given unit area) density was 270 plants m^−2^, and the planting row spacing is 25 cm; other cultivation and management practices were the same as those in typical high-yield fields. The wheat was sown on October 14th in both years. The jointing stages occurred on April 8th and April 12th of the following year, respectively. The heading dates were April 25th and April 27th of the subsequent year, respectively. The harvest periods were both set for June 8nd of the following year.

### 2.3 Sampling and measurements

Sampling schedule: The sampling process was initiated on the first day of the dry–hot wind treatment and subsequently repeated at intervals of three days. For each treatment, sampling was consistently conducted at 14:00 each day. Thirty flag leaves of wheat were randomly selected, promptly frozen in liquid nitrogen, stored at -80°C, and subsequently ground to facilitate the measurement of enzyme activity and hormone content in the wheat flag leaves.

#### 2.3.1 Canopy temperature

Using the infrared thermal imaging camera Testo 890-2 (TESTO, Germany), images were captured on the third day after the start of the DH treatment, during the sunny hours between 9:00 AM and 11:00 AM. Care was taken to ensure that the sky was cloud-free during the shooting. The subjects for photography were selected from areas within each plot where the wheat population exhibited uniform growth conditions, to ensure the accuracy of the thermal imaging data. Images were taken at a 45-degree angle to the horizontal, with the camera positioned 1 meter from the target area. Each treatment was photographed three times. After capturing the images, professional analysis was conducted using Testo IRSoft version 4 software (TESTO, Germany). To obtain temperature data for wheat under different treatments, we manually and randomly selected measurement points on the wheat spikes and leaves in the captured images. Specifically, for each treatment, 20 points on the spikes and leaves were randomly chosen from the images, and the temperature at each point was recorded. These values were then averaged to calculate the overall average temperature of the spikes and leaves.

#### 2.3.2 SPAD and chlorophyll fluorescence parameters

Wheat plants of a similar size and growth vigor were selected for labeling, with 10 plants labeled for each treatment. Leaf measurements were taken using a handheld SPAD-502 (Konica Minolta, Japan) meter, starting from the first day of the DH treatment. Measurements were taken at the center of each leaf; this was conducted three times to obtain an average value, with intervals of three days between each measurement. The chlorophyll fluorescence parameters of the wheat (Fv/Fm and PIabs) were measured using the M-PEA-2 multifunctional plant efficiency analyzer (Hansatech, United Kingdom). Fv/Fm reflects the maximal ability of photosystem II (PSII) to convert absorbed light energy into chemical energy under specific conditions. PIabs serves as a quantitative measure of the actual photochemical energy conversion efficiency of PSII, providing an absolute value for direct comparisons of photosynthetic efficiency across different plants or within the same plant under varying conditions. Measurements were taken on clear mornings between 9:00 and 11:00 a.m., starting from the first day of the DH treatment, with measurements taken every 3 days.

#### 2.3.3 Measurement of photosynthetic gas exchange parameters

During 2021–2022, using the Li-6400 photosynthesis system (LI-COR, United States of America), the net photosynthetic rate (Pn), stomatal conductance (gs), intercellular CO_2_ concentration (Ci), and transpiration rate (Tr) of wheat flag leaves with consistent light exposure and growth direction were measured. Each measurement was repeated three times, starting from the first day of the DH treatment, measurements were taken on clear mornings between 9:00 and 11:00 a.m., with measurements taken every 4 days.

#### 2.3.4 Activities of antioxidant enzymes

SOD activity (Ug^−1^ FW min^−1^) was determined via the nitroblue tetrazolium photoreduction method, POD (Ug^−1^ FW min^−1^) activity was determined via the guaiacol method, and CAT (Ug^−1^ FW min^−1^) activity was assessed via the hydrogen peroxide method (Wu et al., 2017).

#### 2.3.5 Determination of endogenous hormone content

##### 2.3.5.1 JA and SA

The method for extracting endogenous JA and SA hormones referenced the previous literature (Engelberth et al., 2003) and was optimized and modified as follows: The samples were placed in a mortar and ground into powder with liquid nitrogen. An exact amount of 50–100 mg of each sample was weighed and placed into a 2 mL centrifuge tube, followed by the addition of 1000 µL of acetone–citric acid (7/3) extractant, and 20 µL of mixed internal standard was added to the sample and mixed thoroughly; then, another 800 µL of acetone–citric acid (7/3) extractant was added and mixed thoroughly. The mixture was then placed on a temperature-controlled shaker, maintained at 4°C, and shaken in the dark for 3 h. It was then taken out, kept in the dark, and placed in a fume hood to allow the acetone to evaporate completely. Thereafter, 700 µL of petroleum ether was added to the sample in the centrifuge tube, shaken thoroughly, and centrifuged at low temperature. The supernatant was collected, and another 700 µL of petroleum ether was added to the centrifuge tube, shaken thoroughly, and centrifuged again at low temperature, after which the supernatant was collected. The supernatants from the first two steps were combined and evaporated in a vacuum centrifugal concentrator (Eppendorf, Hamburg, Germany). The samples were re-dissolved in 100 µL of HPLC-grade methanol and filtered through a 0.22 µm organic filter and analyze using a Waters Triple Quadrupole Liquid Chromatography–Mass Spectrometry System (UPLC/XEVO TQ-S, Waters Corporation, United States of America) with a 5 µL injection volume.

##### 2.3.5.2 ABA

The method for extracting ABA from wheat flag leaves was optimized and modified as follows (Dave et al., 2011). Samples were placed in a mortar and ground into powder with liquid nitrogen, and an exact amount of 80–100 mg of the sample was weighed into a 2 mL centrifuge tube. Subsequently, 1.2 mL of is propanol–acetic acid (99/1) extractant was added, followed by 20 µL of mixed internal standard, and the mixture was thoroughly combined. After thorough mixing, the centrifuge tubes were placed on a temperature-controlled shaker at 4°C and shaken for 3 h in the dark at a speed of 200 rpm. Subsequently, the mixture was centrifuged in the dark at 13,200 × g for 300 s. The supernatant was then transferred to a new centrifuge tube. Another 0.8 mL of isopropanol–acetic acid (99/1) extractant was added for a 2 h extraction. The supernatants from both extractions were combined and evaporated to dryness in a vacuum centrifugal concentrator (Eppendorf, Hamburg, Germany). The samples were re-dissolved in 100 µL of HPLC-grade methanol and filtered through a 0.22 µm organic filter and analyze using a Waters Triple Quadrupole Liquid Chromatography–Mass Spectrometry System (UPLC/XEVO TQ-S, Waters Corporation, United States of America) with a 5 µL injection volume.

#### 2.3.6 Determination of dry matter partitioning

On the days of DH treatment, and at maturity, 10 wheat stems with consistent growth and size were taken from each plot for each treatment, with 3 replicates, giving a total of 30 wheat stems. The wheat samples on the day of DH treatment were divided into three parts: stem + sheath, leaves, and spikes. The wheat samples at maturity were divided into four parts: grains, rachis + glumes, leaves, and stem + sheath. After sampling, the samples were placed in an oven at 105°C for 30 min to deactivate enzymes and then dried at 70°C to a constant weight before weighing.

DBD represents the dry matter transported before the DH (g stalk^−1^); DMB is the dry matter mass before the DH (g stalk^−1^); DMM refers to the dry matter mass at maturity (g stalk^−1^); DAD is the dry matter transported after the DH (g stalk^−1^); GY stands for grain yield (g stalk^−1^); CBD is the contribution of assimilate transport to grain filling before the DH (%); and CAD is the contribution of assimilate transport to grain filling after the DH (%).


(1) DBD=DMB−DMM(stem+sheath+leaves+glumes).

(2) DAD=GY−DBD.

(3) CBD=DBD/GY×100%.

(4) CAD=DAD/GY×100%.

#### 2.3.7 Yield and yield components

At the wheat maturity stage, field yield measurement was conducted, with 1 m² selected from each plot for yield measurement and three replicates. For each treatment, the number of spikes per unit area and the number of grains per spike were counted on a double row of 1 m. After threshing, the thousand-grain weight and other indicators were measured and recorded.

#### 2.3.8 Statistical analysis and plotting

A three-way ANOVA was conducted using DPS9.01 Statistical Package (Zhejiang University, Hangzhou, China) on the wheat trait as the response variable and the ‘year’, ‘dry-hot wind’ and ‘24-epibrassinolide’ as fixed variables. Multiple comparisons of each indicator under different treatments were performed using the LSD method with a significant probability level of 0.05. All the graphs were plotted using the “ggplot” package in Origin 2020 and R (4.1.1) (R Core Team, Vienna, Austria, 2020). To explore the complex direct and indirect relationships among variables, structural equation models (SEMs) were constructed. SEMs were used to test and estimate causal relationships using a combination of statistical data and qualitative causal assumptions. The models were built using the “lavaan” package (Rosseel, 2012), which is widely used in SEMs for tasks such as confirmatory factor analysis, path analysis, and other types of structural model estimation.

## 3 Results

### 3.1 Endogenous hormone

**Figure 2:**
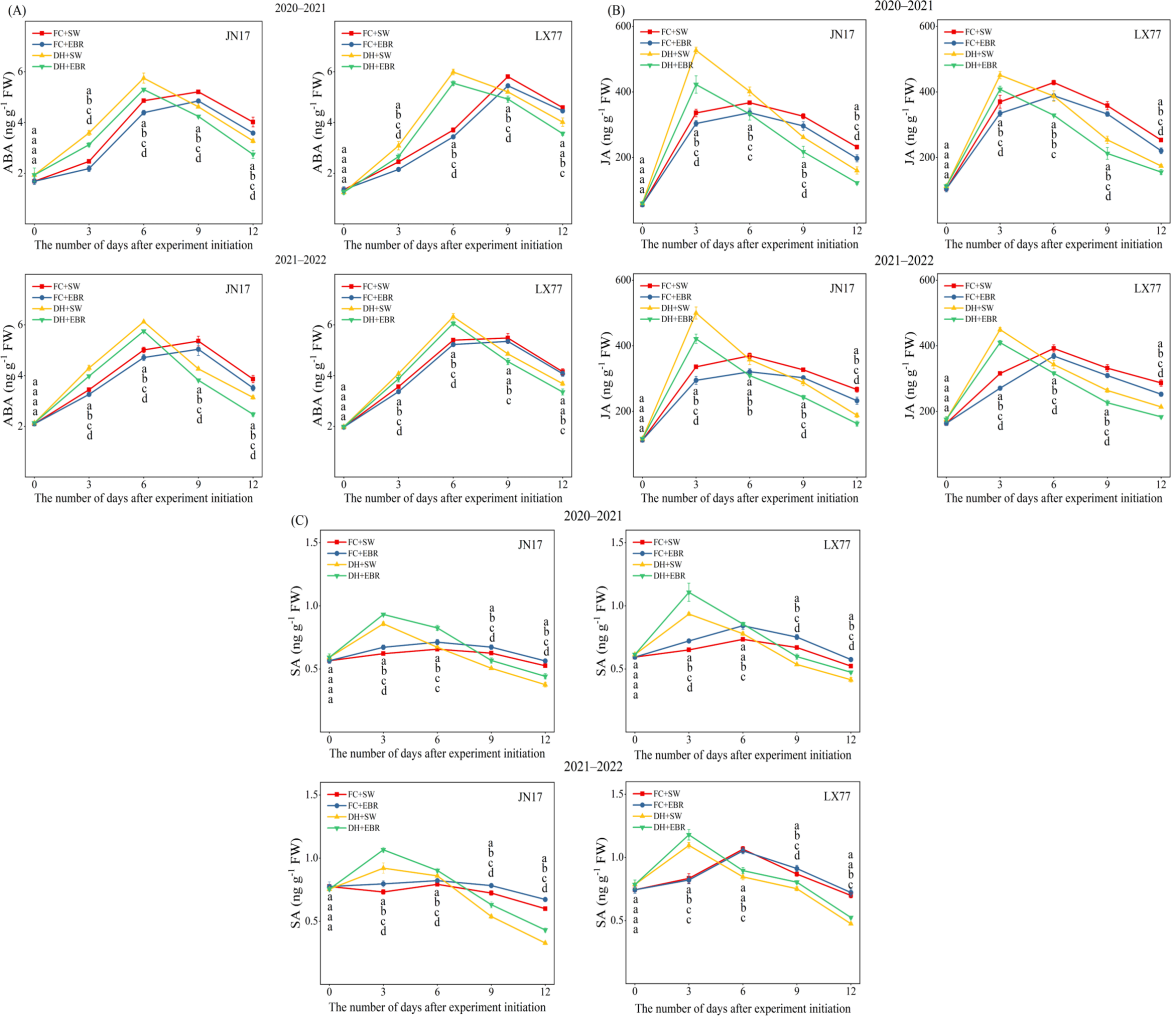
Endogenous hormone regulation of wheat flag leaves in dry–hot wind conditions. **(A)** Abscisic acid (ABA); **(B)** jasmonic acid (JA); **(C)** salicylic acid (SA). FC + SW, FC + EBR, DH + SW, and DH + EBR, respectively, represent spraying water under field conditions, spraying 24-epibrassinolide under field conditions, spraying water under dry–hot wind conditions, and spraying 24-epibrassinolide under dry–hot wind conditions. Different letters indicate significant differences at the 0.05 level (*p*< 0.05). Bars represent mean values ± SE (n = 3).

The DH and EBR have a significant effect on the endogenous hormone content in the flag leaves of wheat (*p*< 0.01) (**Table 1**). The ABA content in wheat flag leaves showed a trend of first increasing and then decreasing, with more pronounced changes under DH treatment. Among the measured time points, ABA content under DH conditions reached its maximum on the sixth day, while it peaked on the eighth day under FC conditions. Compared to SW treatment, EBR significantly reduced ABA content in JN17 and LX77 under DH conditions, by 6% to 21% and 4% to 14%, respectively. In contrast, under FC conditions, ABA levels decreased by 5.5% to 11.5% and 1.9% to 12.1%, respectively (**Figure 2A**). Compared to FC, the reduction in ABA content was more pronounced under DH conditions with EBR treatment. Similarly, JA content also exhibited a trend of first increasing and then decreasing. Under DH conditions, JA content increased during the early stage of treatment and peaked on the third day of the measurement period. Compared to SW treatment, EBR treatment reduced JA content in JN17 and LX77 under DH conditions by 13.4% to 23.4% and 7.7% to 16.4%, respectively. Under FC conditions, EBR treatment decreased JA content in JN17 by 6.8% to 14.8%, while the reduction in LX77 ranged from 5.8% to 14.1% (**Figure 2B**).

**Table 1:** The significant effects (*p* values) of dry–hot wind and EBR spraying on hormone levels and antioxidant enzyme activity in different wheat cultivars in 2020 and 2021.

Terms	Y	C	D	E	Y×C	Y×D	Y×E	C×D	C×E	D×E	Y×C×D	Y×C×E	Y×D×E	C×D×E	Y×C×D×E
ABA	**	ns	**	**	**	**	*	**	ns	*	**	ns	ns	ns	ns
JA	**	**	**	**	*	**	ns	**	**	**	**	ns	ns	**	ns
SA	**	**	**	**	ns	ns	ns	**	ns	**	ns	**	ns	ns	ns
SOD	**	ns	**	**	**	**	ns	**	ns	**	ns	ns	ns	ns	*
POD	**	ns	**	**	**	ns	ns	**	*	**	ns	ns	ns	ns	ns
CAT	**	**	**	**	ns	*	ns	*	ns	**	ns	**	ns	ns	ns

Y: year; C: cultivar; D: dry–hot wind; E: 24-epibrassinolide; ABA: abscisic acid; JA: jasmonic acid; SA: salicylic acid; SOD: superoxide dismutase; POD: peroxidase; CAT: catalase; *P<0.05; **P<0.01; ns: *P*>0.05.

SA content rapidly increased during the early phase of treatment. SA content peaked on the third day of the measurement period under DH conditions, whereas it peaked on the sixth day under FC conditions. Compared to SW, under DH conditions, EBR treatment significantly increased the SA content at all measurement time points following DH treatment. In JN17, the increase ranged from 4.9% to 31.5%, while in LX77, it ranged from 5.8% to 18.4%. Under FC conditions, EBR treatment resulted in a 7.2% to 12.2% increase in SA content in JN17, but significantly affected LX77 only on day 9 after the DH treatment (**Figure 2C**).

### 3.2 Photosynthetic physiological parameters in wheat flag leaves

**Figure 3:**
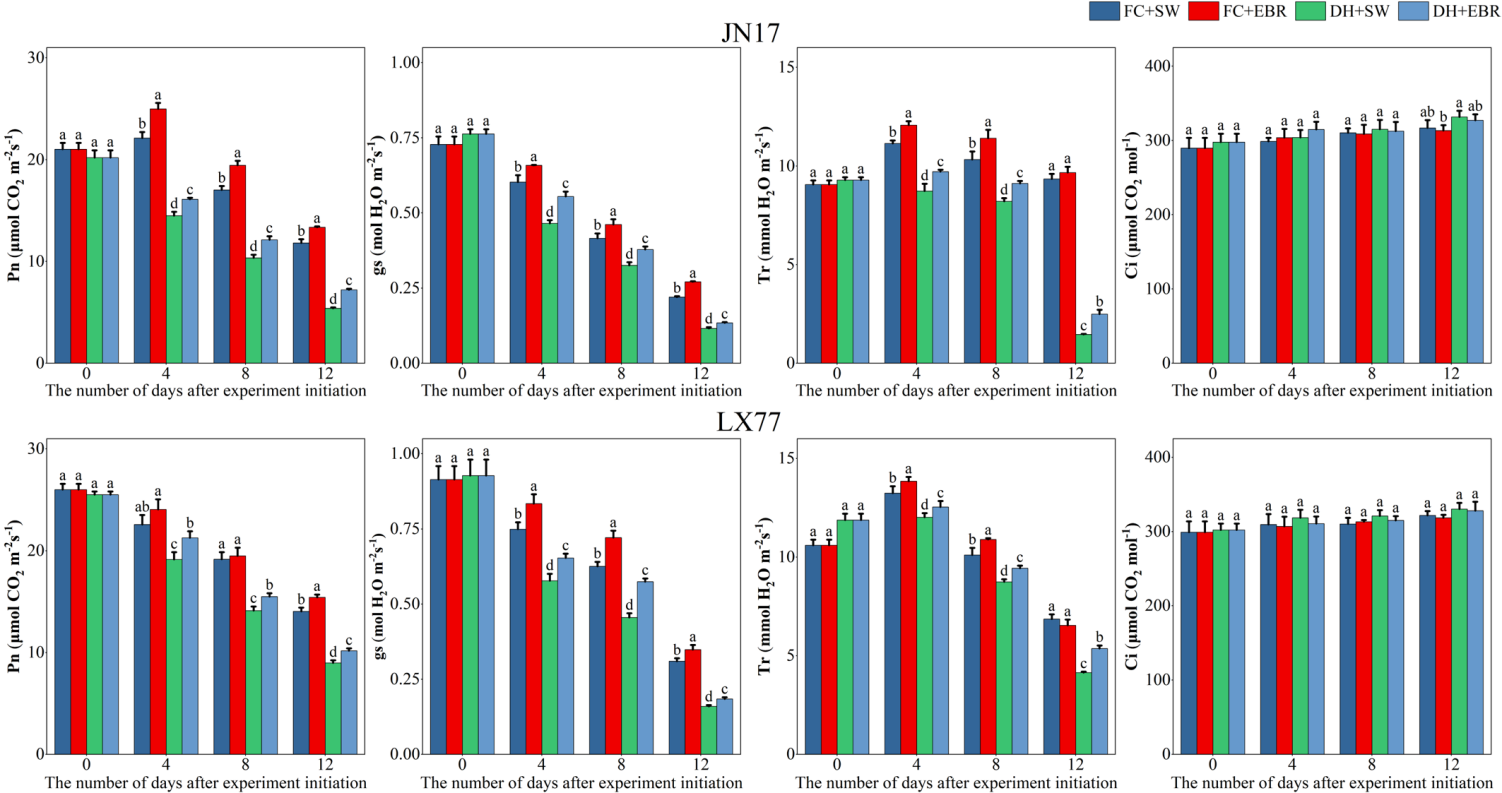
Photosynthesis regulation of wheat flag leaves in dry–hot wind conditions. Pn: net photosynthetic rate; gs: stomatal conductance; Tr: transpiration rate; Ci: intercellular CO_2_ concentration. FC + SW, FC + EBR, DH + SW, and DH + EBR, respectively, represent spraying water under field conditions, spraying 24-epibrassinolide under field conditions, spraying water under dry–hot wind conditions, and spraying 24-epibrassinolide under dry–hot wind conditions. Different letters indicate significant differences at the 0.05 level (*p*< 0.05). Bars represent mean values ± SE (n =3).

The photosynthetic physiological parameters of wheat flag leaves are presented in **Figure 3**. Under DH conditions, the Pn, gs, and Tr of flag leaves in the wheat cultivars JN17 and LX77 were significantly lower compared to the FC environment. A declining trend in Pn, gs, and Tr was observed for both cultivars as the treatment progressed. Compared to SW treatment, under DH conditions, EBR significantly increased Pn, gs, and Tr at all measurement time points after DH treatment in both JN17 and LX77. Under FC conditions, it significantly increased Pn and gs at all measurement time points after the start of the Dh treatment, as well as Tr on days 4 and 8. Treatment had no significant statistical effect on Ci in the wheat flag leaf.

### 3.3 Temperatures of wheat spike and leaf

**Figure 4:**
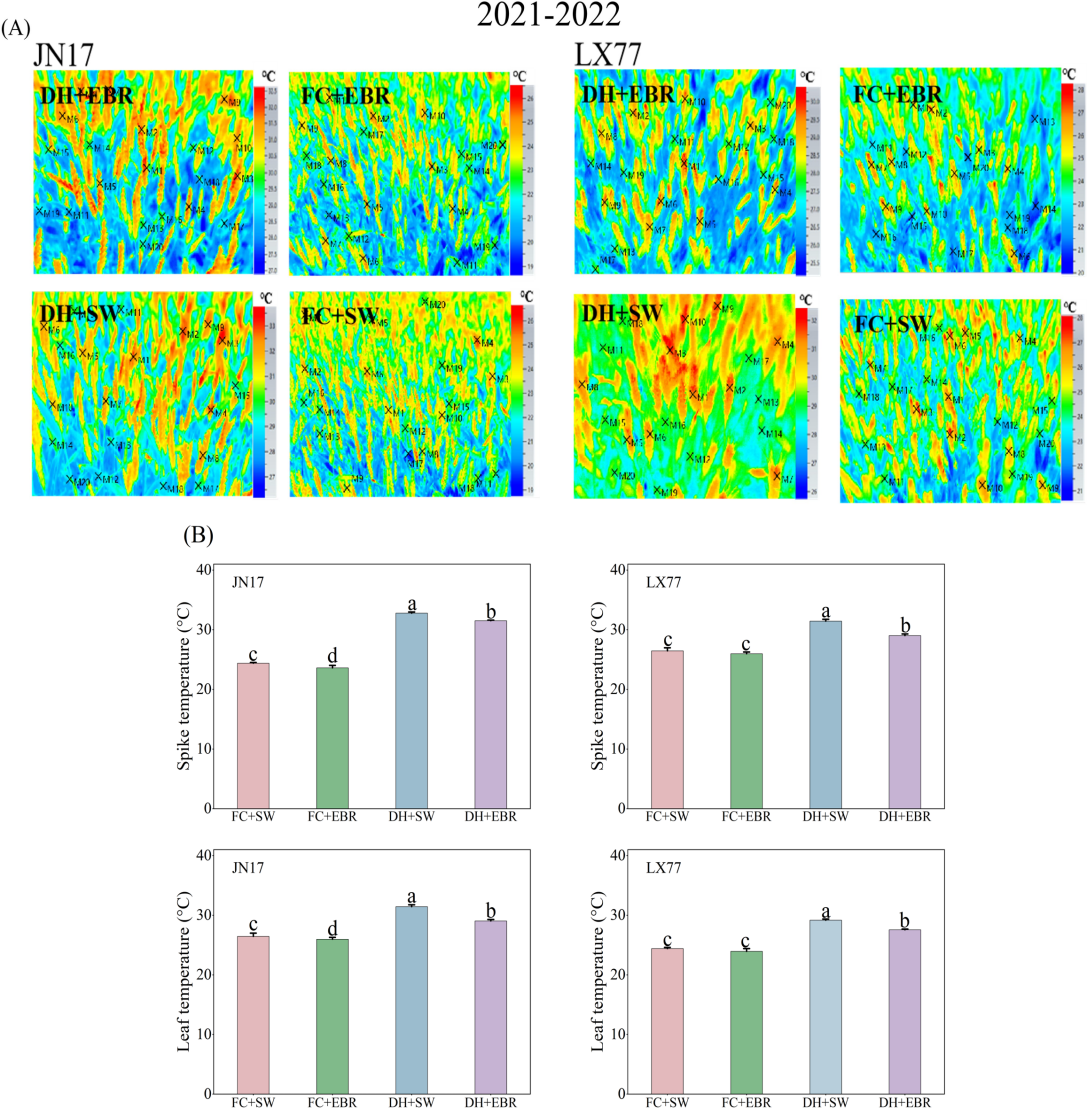
Temperature regulation of wheat spikes and leaves in dry–hot wind conditions. **(A)** Infrared thermograms (×M represents the specific point selected by the software for image data processing); **(B)** the spike and leaf temperatures under different treatments. FC + SW, FC + EBR, DH + SW, and DH + EBR, respectively, represent spraying water under field conditions, spraying 24-epibrassinolide under field conditions, spraying water under dry–hot wind conditions, and spraying 24-epibrassinolide under dry–hot wind conditions. Different letters indicate significant differences at the 0.05 level (*p*< 0.05). Bars represent mean values ± SE (n = 3).

Compared to JN17, the spike and leaf temperatures of the wheat cultivar LX77 were less affected by DH (**Figure 4**). Under DH conditions, EBR significantly reduced spike and leaf temperatures in both cultivars compared to the SW treatment (**Figure 4B**). Specifically, the spike and leaf temperatures of JN17 decreased by 1.27°C and 2.4°C, respectively, while those of LX77 decreased by 0.87°C and 1.6°C, respectively. Under FC conditions, a significant difference was observed in the spike and leaf temperatures of JN17 between the EBR and SW treatments, whereas no statistically significant changes were observed in the spike and leaf temperatures of the wheat cultivar LX77.

### 3.4 Antioxidant enzyme activity in wheat flag leaves

**Figure 5:**
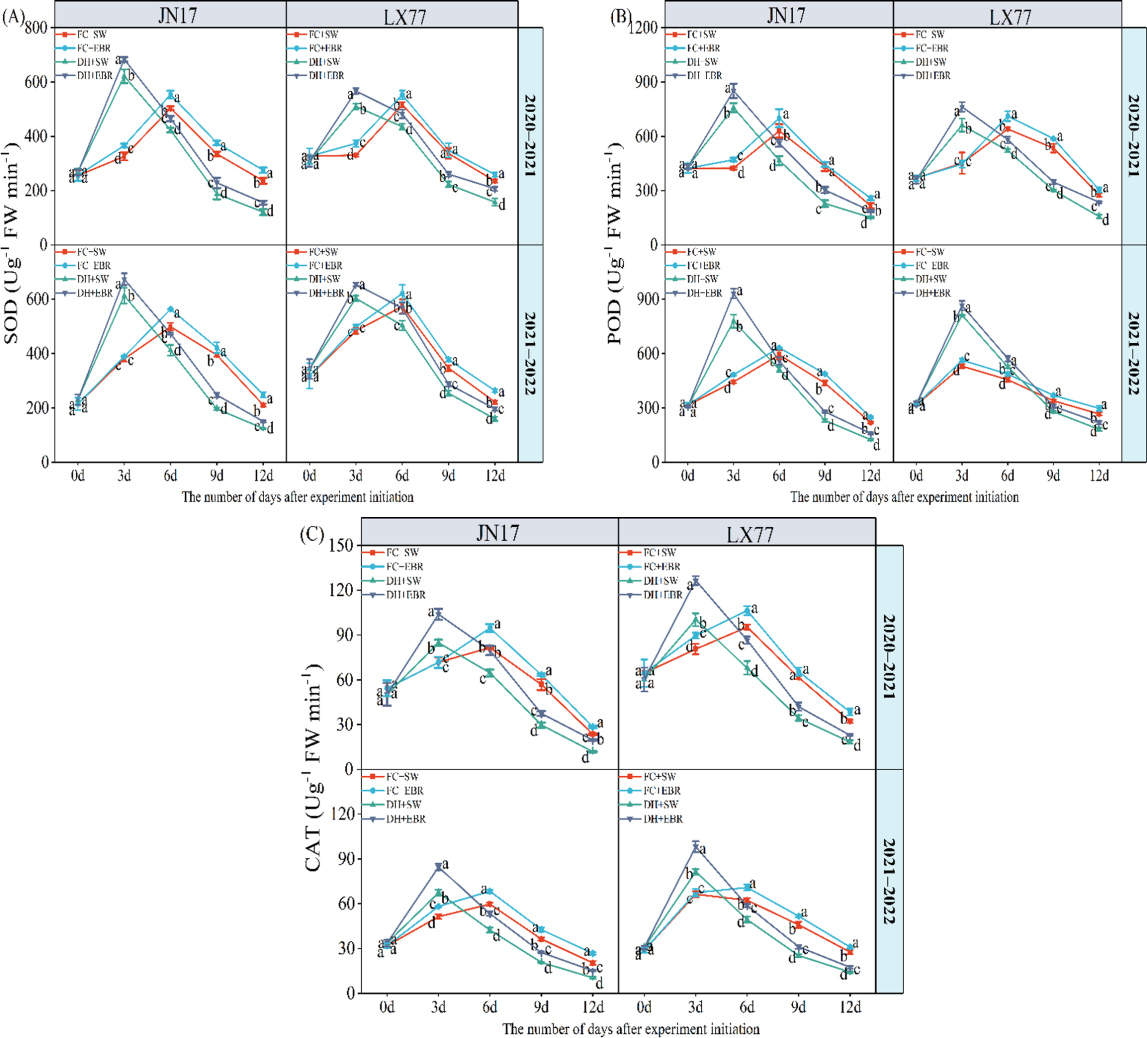
Antioxidant enzyme activity regulation of wheat flag leaves in dry–hot wind conditions. **(A)** Superoxide dismutase (SOD); **(B)** peroxidase (POD); **(C)** catalase (CAT). FC + SW, FC + EBR, DH + SW, and DH + EBR, respectively, represent spraying water under field conditions, spraying 24-epibrassinolide under field conditions, spraying water under dry–hot wind conditions, and spraying 24-epibrassinolide under dry–hot wind conditions. Different letters indicate significant differences at the 0.05 level (*p*< 0.05). Bars represent mean values ± SE (n = 3).

The DH and EBR have a significant effect on the antioxidant enzyme activity in the flag leaves of wheat (*p*< 0.01) (**Table 1**). Under DH treatment, the activities of SOD, POD, and CAT in the flag leaves of the JN17 and LX77 wheat cultivars initially showed an increasing trend, followed by a subsequent decrease. Specifically, during the early stages of treatment, SOD activity under DH conditions was significantly higher than FC environment (**Figure 5A**). However, as the stress persisted, SOD activity in the wheat under DH conditions significantly decreased in the middle and later stages of treatment. During the early stages of DH stress, POD and CAT activities exhibited similar patterns (**Figures 5B, C**). EBR significantly increased the content of three antioxidant enzymes under DH conditions.

### 3.5 SPAD in wheat flag leaves

**Figure 6:**
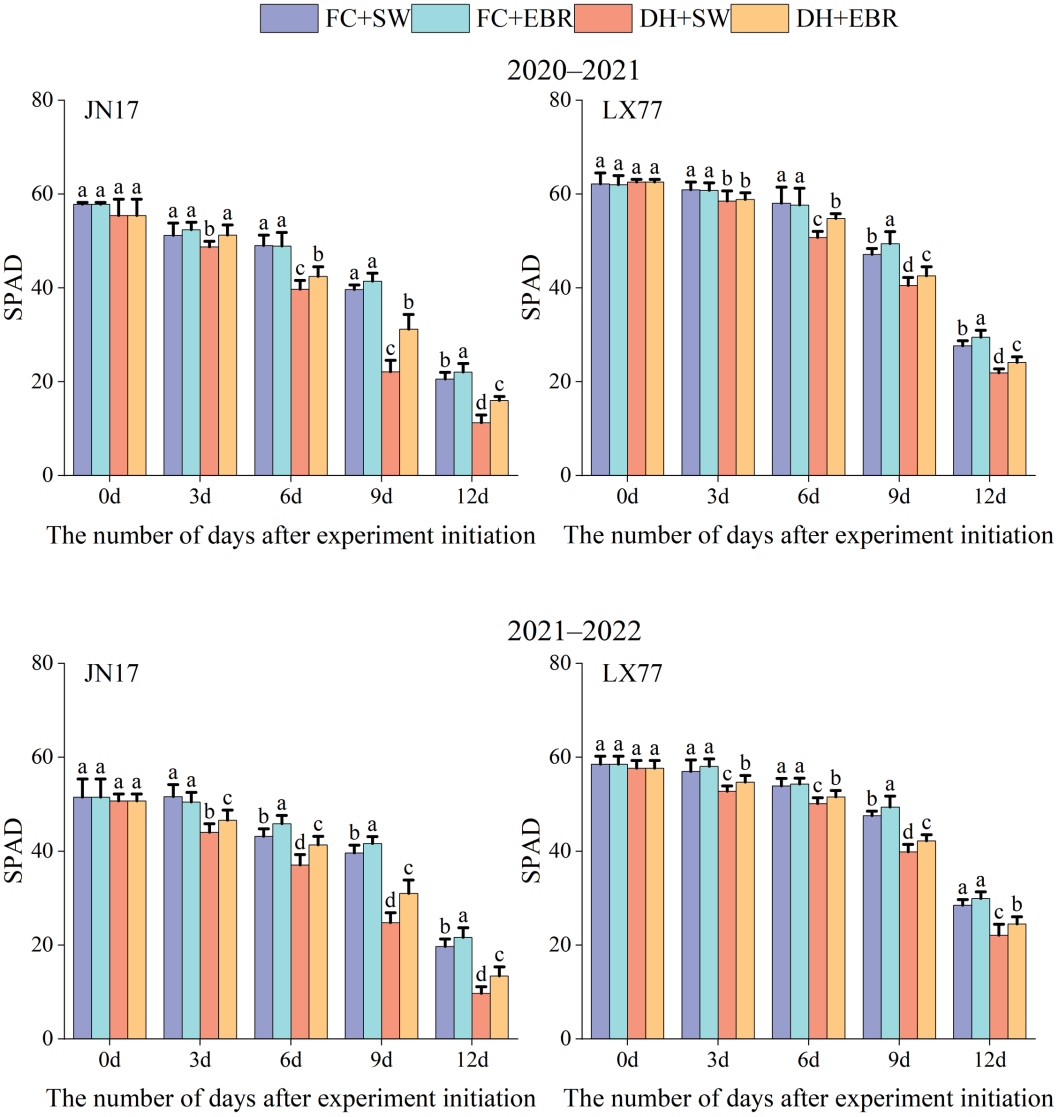
SPAD values regulation of wheat flag leaves in dry-hot wind conditions. FC + SW, FC + EBR, DH + SW, and DH + EBR, respectively, represent spraying water under field conditions, spraying 24-epibrassinolide under field conditions, spraying water under dry–hot wind conditions, and spraying 24-epibrassinolide under dry–hot wind conditions. Different letters indicate significant differences at the 0.05 level (*p*< 0.05). Bars represent mean values ± SE (n = 10).

As shown in **Figure 6**, the SPAD values of flag leaves in the JN17 and LX77 exhibited a declining trend after the initiation of the DH treatment. Initially, the SPAD values of flag leaves under DH conditions were comparable to those under FC conditions. However, as the duration of the DH treatment increased, a noticeable difference was observed between the SPAD values under DH and FC conditions. Under DH conditions, the SPAD values in the EBR treatment were significantly higher than those in the SW treatment. Under FC conditions, there was no significant difference in SPAD values between the EBR and SW treatments at the early stages of the experimental treatment. As the experiment progressed, the SPAD values in the EBR treatment became significantly higher than those in the SW treatment.

### 3.6 Fluorescence characteristics in wheat flag leaves

**Figure 7:**
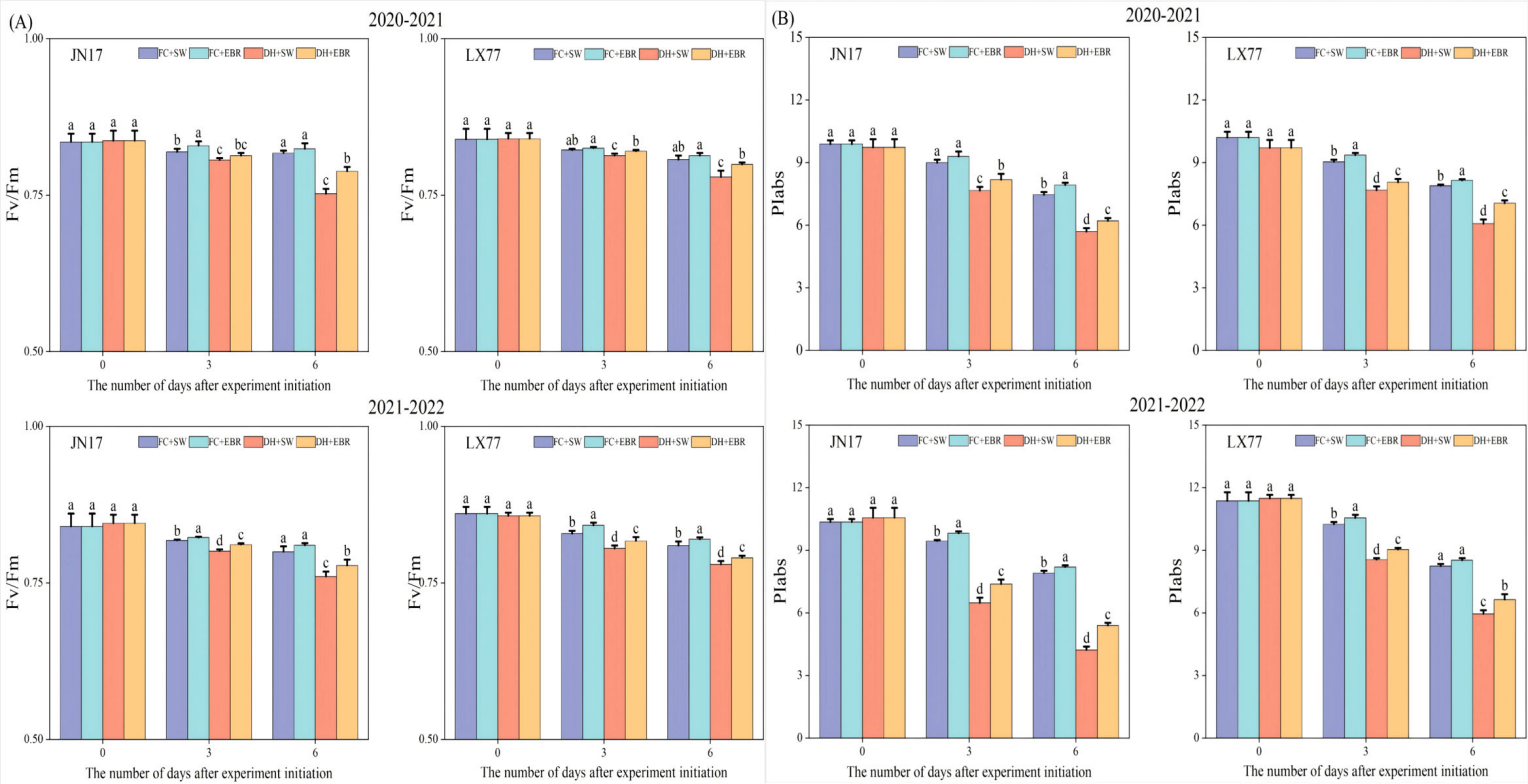
Fluorescence characteristics regulation of wheat flag leaves in dry-hot wind conditions. **(A)** The maximal photochemical efficiency of the photosystem II (Fv/Fm). **(B)** Photosynthetic performance index based on absorption (PIabs). FC + SW, FC + EBR, DH + SW, and DH + EBR, respectively, represent spraying water under field conditions, spraying 24-epibrassinolide under field conditions, spraying water under dry–hot wind conditions, and spraying 24-epibrassinolide under dry–hot wind conditions. Different letters indicate significant differences at the 0.05 level (*p*< 0.05). Bars represent mean values ± SE (n = 3).

The maximal photochemical efficiencies of the PSII (Fv/Fm) in the flag leaves of both wheat cultivars grown under DH conditions were significantly lower than those of FC-grown wheat (**Figure 7A**). Notably, the LX77 flag leaves exhibited less sensitivity to the detrimental effects of DH stress on Fv/Fm values than JN17, suggesting that photosynthesis in LX77 was less impacted as well. Under DH conditions, the application of EBR significantly elevated the Fv/Fm values in the flag leaves of both wheat cultivars compared to SW. Moreover, during the course time of the treatments, the disparity in the Fv/Fm values between SW- and EBR-treated wheat leaves widened, indicating that EBR can effectively mitigate the adverse effects of DH stress on PSII photochemical efficiency.

Similarly, photosynthetic performance indices based on the absorption (PIabs) of the flag leaves of both wheat cultivars under DH conditions significantly varied compared to the FC-grown wheat (**Figure 7B**). The PIabs of LX77 showed less fluctuation than that of JN17 under DH conditions, indicating a relatively more stable photosynthetic performance. Furthermore, PIabs exhibited greater variability and a faster rate of decline with the increasing duration of DH treatment compared to Fv/Fm. Under DH conditions, the EBR treatment significantly enhanced the PIabs values in the flag leaves of both wheat cultivars compared to SW.

### 3.7 Dry matter accumulation and distribution

Prior to DH treatment, the dry matter weights and organ proportions of the JN17 and LX77 wheat cultivars were comparable, as shown in **Table 2**. Significant reductions in the dry matter weights of the stem + sheath and leaf at maturity were observed in both cultivars following the DH treatment, but there was no significant impact on the rachis +glumes (**Table 3**). Specifically, under DH conditions, the total dry matter weight of JN17 decreased by 16%, the stem + sheath weight decreased by 8%, the leaf weight decreased by 25%, and the grain weight decreased by 23.1%, whereas LX77 showed reductions of 13.5%, 6.2%, 22.5%, and 18.6%, respectively, compared to the FC conditions.

**Table 2:** Weights and proportions of dry matter in different wheat organs at the beginning dry–hot wind treatment.

Cultivar	Treatment	Total (g·Stalk^−1^)	Stem + Sheath		Leaf		Spike	
			Weight (g·Stalk^−1^)	Ratio (%)	Weight (g·Stalk^−1^)	Ratio (%)	Weight (g·Stalk^−1^)	Ratio (%)
JN17	FC + SW	1.98 ± 0.12a	1.15 ± 0.06a	58.0 a	0.32 ± 0.02a	16.3 a	0.51 ± 0.04a	25.7 a
	FC + EBR	2.03 ± 0.05a	1.12 ± 0.03a	55.1 a	0.34 ± 0.01a	16.9 a	0.57 ± 0.02a	28.0 a
	DH + SW	1.96 ± 0.07a	1.07 ± 0.05a	59.4 a	0.32 ± 0.03a	16.7 a	0.52 ± 0.02a	27.4 a
	DH + EBR	1.94 ± 0.08a	1.10 ± 0.03a	56.8 a	0.31 ± 0.02a	15.9 a	0.53 ± 0.04a	27.3 a
LX77	FC + SW	2.29 ± 0.05a	1.37 ± 0.05a	59.8 a	0.33 ± 0.03a	14.5 a	0.59 ± 0.07a	25.7 a
	FC + EBR	2.30 ± 0.09a	1.37 ± 0.10a	59.4 a	0.35 ± 0.03a	15.2 a	0.58 ± 0.04a	25.4 a
	DH + SW	2.23 ± 0.06a	1.34 ± 0.05a	60.2 a	0.33 ± 0.03a	14.9 a	0.55 ± 0.05a	24.9 a
	DH + EBR	2.17 ± 0.02a	1.33 ± 0.03a	61.3 a	0.33 ± 0.03a	15.1 a	0.51 ± 0.12a	23.6 a

FC + SW, FC + EBR, DH + SW, and DH + EBR, respectively, represent spraying water under field conditions, spraying 24-epibrassinolide under field conditions, spraying water under dry–hot wind conditions, and spraying 24-epibrassinolide under dry–hot wind conditions. Different letters indicate significant differences at the 0.05 level (*p* < 0.05). “Ratio” represents the proportion of the dry matter weight of the organ to the total dry matter weight of the whole plant.

**Table 3:** Weights and proportions of dry matter in different organs of wheat at maturity.

Cultivar	Treatment	Total (g·Stalk^−1^)	Stem + Sheath		Leaf		Rachis + Glumes		Grain	
			Weight (g·Stalk^−1^)	Ratio (%)	Weight (g·Stalk^−1^)	Ratio (%)	Weight (g·Stalk^−1^)	Ratio (%)	Weight (g·Stalk^−1^)	Ratio (%)
JN17	FC + SW	2.80 ± 0.03b	0.87 ± 0.01a	31.2 c	0.25 ± 0.01b	9.0 a	0.37 ± 0.01a	13.1 c	1.31 ± 0.04b	46.8 ab
	FC + EBR	3.00 ± 0.04a	0.90 ± 0.02a	29.9 d	0.27 ± 0.01a	8.9 a	0.37 ± 0.01a	12.4 d	1.46 ± 0.03a	48.8 a
	DH + SW	2.33 ± 0.04d	0.80 ± 0.01c	34.5 a	0.17 ± 0.01d	7.1 b	0.36 ± 0.01a	15.6 a	1.00 ± 0.03d	42.9 c
	DH + EBR	2.54 ± 0.07c	0.83 ± 0.01b	32.6 b	0.22 ± 0.01c	8.8 a	0.36 ± 0.02a	14.3 b	1.13 ± 0.05c	44.3 bc
LX77	FC + SW	3.10 ± 0.06b	0.96 ± 0.01ab	30.0 c	0.27 ± 0.01b	8.8 b	0.42 ± 0.01a	13.5 b	1.45 ± 0.06b	46.9 ab
	FC + EBR	3.29 ± 0.07a	0.98 ± 0.02a	29.7 d	0.31 ± 0.01a	9.5 a	0.40 ± 0.01a	12.2 c	1.60 ± 0.08a	48.6 a
	DH + SW	2.66 ± 0.01d	0.89 ± 0.03c	33.8 a	0.21 ± 0.02d	7.8 c	0.39 ± 0.01a	14.8 a	1.17 ± 0.03d	44.1 b
	DH + EBR	2.87 ± 0.05c	0.93 ± 0.01b	31.5 b	0.24 ± 0.01c	8.3 bc	0.39 ± 0.01a	13.8 b	1.31 ± 0.05c	45.8 ab

FC + SW, FC + EBR, DH + SW, and DH + EBR, respectively, represent spraying water under field conditions, spraying 24-epibrassinolide under field conditions, spraying water under dry–hot wind conditions, and spraying 24-epibrassinolide under dry–hot wind conditions. Different letters indicate significant differences at the 0.05 level (*p*< 0.05). “Ratio” represents the proportion of the dry matter weight of the organ to the total dry matter weight of the whole plant.

Remarkably, EBR mitigated these reductions, significantly enhancing organ dry matter weights under DH treatment, as is evident in **Table 3**, compared to SW, EBR increased the total dry matter weights by 9% in JN17 and 7.9% in LX77, stem + sheath weights by 3.75% and 4.49%, leaf weights by 29.4% and 14.3%, and grain weights by 13% and 11.9%, respectively. No significant effects on the dry matter weights of rachis + glumes were observed. In addition, EBR regulated organ dry matter weight as a proportion of the whole plant in both varieties, reducing the proportion of stems + sheaths and increasing the proportion of grains (**Table 4**).

**Table 4:** Redistribution of dry matter in vegetative organs of wheat after dry–hot wind treatment.

Cultivar	Treatment	DBD (g Stalk^−1^)	DAD (g Stalk^−1^)	CBD (%)	CAD (%)
JN17	FC + SW	0.50 ± 0.12a	0.82 ± 0.15a	37.8 c	62.2 b
	FC + EBR	0.50 ± 0.07a	0.97 ± 0.09a	33.9 d	66.2 a
	DH + SW	0.63 ± 0.07a	0.37 ± 0.06c	62.6 a	37.4 d
	DH + EBR	0.53 ± 0.09a	0.60 ± 0.10b	47.0 b	53.1 c
LX77	FC + SW	0.65 ± 0.06ab	0.80 ± 0.04b	44.8 b	55.2 b
	FC + EBR	0.61 ± 0.10b	1.00 ± 0.17a	37.9 c	62.1 a
	DH + SW	0.74 ± 0.03a	0.43 ± 0.06c	63.3 a	36.7 c
	DH + EBR	0.62 ± 0.03ab	0.70 ± 0.06b	46.9 b	53.1 b

FC + SW, FC + EBR, DH + SW, and DH + EBR, respectively, represent spraying water under field conditions, spraying 24-epibrassinolide under field conditions, spraying water under dry–hot wind conditions, and spraying 24-epibrassinolide under dry–hot wind conditions. Different letters indicate significant differences at the 0.05 level (*p* < 0.05). “Ratio” represents the proportion of the dry matter weight of the organ to the total dry matter weight of the whole plant.

### 3.8 Yield and yield components

DH stress significantly impacted wheat grain yield, as shown in **Table 5**. Compared to wheat grown under FC conditions, JN17 experienced a yield reduction of approximately 11.8% in the first year and 9.7% in the second year under DH conditions. Similarly, LX77 showed a yield reduction of around 9% in the first year and 6.6% in the second year under the same conditions. Notably, DH stress did not significantly alter the number of spikes per unit area or the number of grains per spike, but it significantly decreased wheat thousand-grain weight (**Table 5**). Specifically, compared to FC, JN17 exhibited a reduction in thousand-grain weight of approximately 12.1% in the first year and 9% in the second year, while LX77 showed reductions of about 6.9% and 6.3%, respectively. Analysis revealed that LX77’s yield and thousand-grain weight were less affected by DH stress compared to JN17.

**Table 5:** EBR regulates wheat yield and its components under dry–hot wind conditions.

Years	Cultivar	Treatment	Spike number (m^-2^)	Grain number per spike	1000-grain weight (g)	Grain yield (kg ha^-1^)
2020–2021	JN17	FC + SW	609.00 ± 12.17a	43.67 ± 2.08a	41.52 ± 1.11b	7195.23 ± 76.10b
		FC + EBR	607.33 ± 11.02a	44.00 ± 1.00a	43.15 ± 0.69a	7564.98 ± 47.88a
		DH + SW	602.67 ± 9.97a	44.33 ± 1.53a	36.34 ± 0.56d	6222.41 ± 107.50d
		DH + EBR	614.33 ± 19.86a	44.00 ± 2.65a	38.12 ± 0.87c	6793.33 ± 78.29c
	LX77	FC + SW	600.33 ± 12.50a	43.33 ± 1.53a	44.30 ± 0.31a	8092.85 ± 136.05a
		FC + EBR	603.00 ± 4.58a	45.00 ± 1.00a	44.94 ± 0.49a	8173.87 ± 104.04a
		DH + SW	605.67 ± 12.66a	44.67 ± 3.06a	41.06 ± 0.49c	7221.55 ± 92.86c
		DH + EBR	601.00 ± 13.11a	44.33 ± 1.53a	42.04 ± 0.56b	7577.86 ± 78.23b
2021–2022	JN17	FC + SW	647.00 ± 10.83a	41.00 ± 1.00a	41.26 ± 0.18b	7326.30 ± 84.55b
		FC + EBR	651.00 ± 14.24a	40.67 ± 2.08a	42.80 ± 0.70a	7644.16 ± 75.14a
		DH + SW	645.87 ± 22.21a	41.00 ± 2.00a	37.09 ± 0.63d	6515.48 ± 27.69d
		DH + EBR	636.64 ± 7.08a	41.33 ± 1.53a	39.43 ± 1.04c	7002.71 ± 51.19c
	LX77	FC + SW	653.00 ± 32.20a	40.00 ± 1.00a	42.50 ± 0.90a	8373.86 ± 74.50a
		FC + EBR	649.00 ± 16.25a	41.00 ± 1.00a	43.13 ± 0.41a	8457.33 ± 47.01a
		DH + SW	645.00 ± 15.57a	39.67 ± 1.53a	39.33 ± 0.51c	7773.60 ± 124.63c
		DH + EBR	641.00 ± 23.27a	40.67 ± 1.53a	40.89 ± 0.71b	7951.14 ± 69.53b
Analysis of variance			*F* values			
Year (Y)			77.80**	46.08**	9.59**	120.60**
Cultivars (C)			0.18	0.10	129.76**	1345.74**
Dry–hot wind (D)			0.56	0.10	325.51**	827.81**
EBR (E)			0.02	0.65	46.78**	148.51**
Y×C			0.70	0.94	23.98**	15.01**
Y×D			0.93	0.10	4.41*	10.60**
Y×E			0.33	0.03	0.40	2.42
C×D			0.01	0.10	14.72**	9.65**
C×E			0.16	0.65	4.58*	27.27**
D×E			0.04	0.24	1.86	13.61**
Y×C×D			0.08	0.10	1.45	0.12
Y×C×E			0.06	0.03	0.01	0.04
Y×D×E			0.27	0.65	0.59	1.13
C×D×E			0.04	0.24	0.04	0.01
Y×C×D×E			215.35	0.08*	0.01**	4185.8145

FC + SW, FC + EBR, DH + SW, and DH + EBR, respectively, represent spraying water under field conditions, spraying 24-epibrassinolide under field conditions, spraying water under dry–hot wind conditions, and spraying 24-epibrassinolide under dry–hot wind conditions. Different letters indicate significant differences at the 0.05 level (*p*< 0.05). *p<0.05; **p<0.01.

EBR significantly increased wheat yield and thousand-grain weight, with no significant effects on the number of spikes per unit area or grains per spike. Under DH conditions, compared to SW, EBR elevated the yield of JN17 by approximately 9.2% in the first year and 7.5% in the second year; the yield of LX77 increased by 4.9% and 2.3%, respectively. In contrast, under FC conditions, EBR increased the yield of JN17 by 5.1% and 4.3% in the first and second years, respectively, while the yield of LX77 was enhanced by about 1% in both years. Importantly, EBR’s enhancing effect on wheat yield under DH stress was markedly more pronounced than under FC conditions.

### 3.9 Analysis of correlation

**Figure 8:**
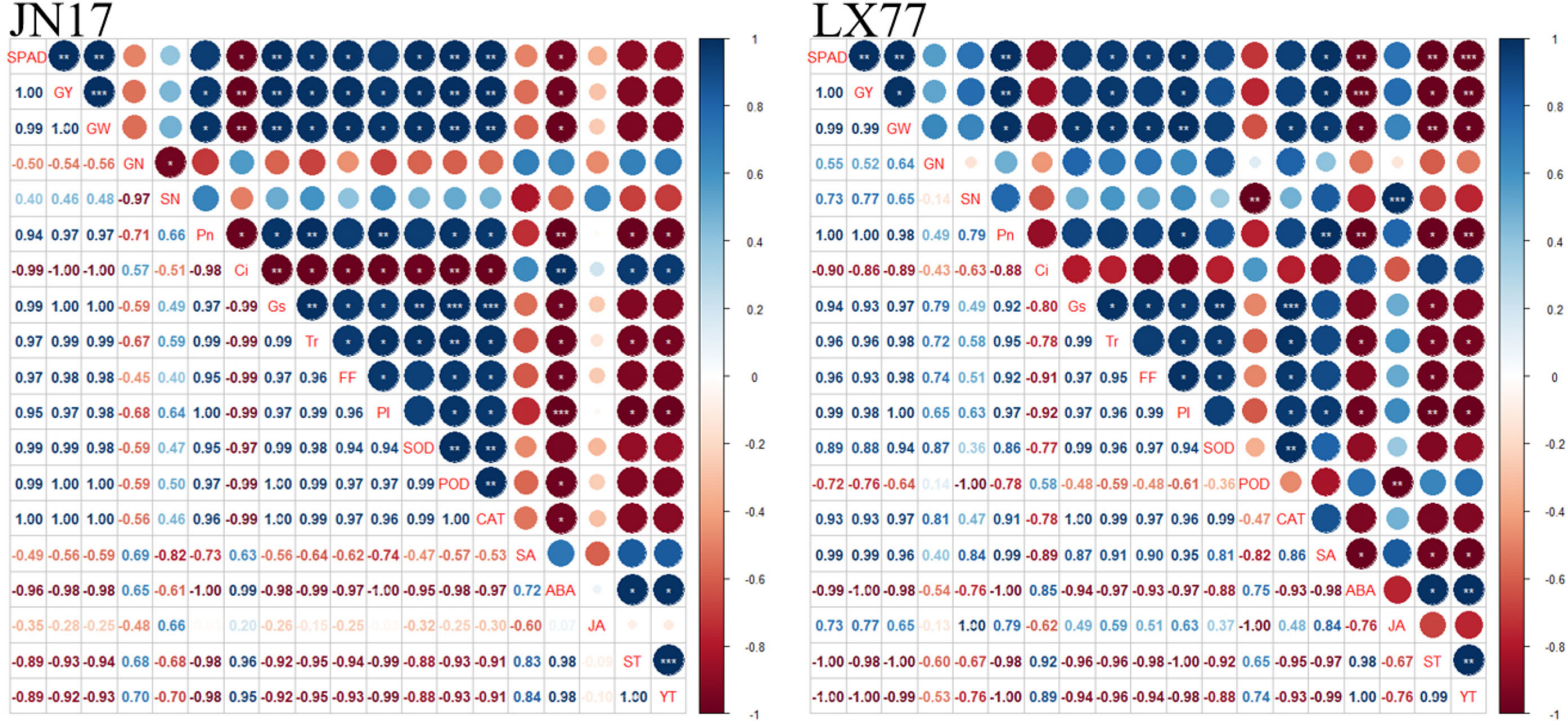
Correlation analysis. GY represents yield (kg ha^-1^); GW represents 1000-grain weight (g); GN represents grain number (Spike^-1^); SN represents spike number (plants m^-2^); FF represents Fv/Fm; PI represents PIabs; ST represents spike temperature (℃); and YT represents leaf temperature (℃). Pn represents net photosynthetic rate; Ci represents lntercellular CO_2_ concentration; gs represents stomatal conductance; Tr represents transpiration rate; SOD represents superoxide dismutase; POD represents peroxidase; CAT represents catalase. SA represents salicylic acid; ABA represents abscisic acid; JA represents jasmonic acid. * represents a significant correlation (*p<* 0.05), ** represents a fairly significant correlation (*p*< 0.01), and *** represents a very significant correlation (*p*< 0.001). The darker the color, the stronger the correlation (blue indicates a positive correlation and red indicates a negative correlation).

A Pearson’s correlation analysis was conducted on the experimental measurement indicators (**Figure 8**). The ABA content in the flag leaves of the JN17 and LX77 wheat varieties exhibited a significant negative correlation with gs. ABA content was significantly negatively correlated with both yield and Pn in these two wheat varieties. The gs of flag leaves in JN17 and LX77 was significantly positively correlated with Tr. Conversely, gs was negatively correlated with spike temperature and leaf temperature. Spike temperature and leaf temperature were negatively correlated with the activities of antioxidant enzymes, including SOD and CAT, as well as with Fv/Fm. Notably, spike temperature and leaf temperature were significantly negatively correlated with PIabs of wheat flag leaves. Furthermore, spike temperature and leaf temperature were negatively correlated with yield in JN17 and significantly negatively correlated with yield in LX77. In summary, exogenous EBR enhanced wheat’s resistance to DH, particularly by reducing spike and leaf temperatures, optimizing hormonal balance in the leaves, and increasing antioxidant capacity. This was beneficial for improving the photosynthetic capacity of wheat leaves under DH conditions, thereby promoting dry matter accumulation and maintaining yield.

### 3.10 Structural equation modeling

Under the SW treatment, the model results showed a good fit (χ^2^ = 3.159, df = 3.000, GFI = 1.000, RMSEA = 0.047, p = 0.368), and all fit indices indicated that the data matched the model well. This model adequately explained the latent variables and their related path coefficients, revealing the effects of different factors on Pn. The positive path coefficient between leaf temperature (T) and ABA was 0.97 (*p*< 0.001), indicating that under SW treatment, an increase in T significantly elevated ABA content. Similarly, T had a significant negative effect on SOD activity, with a path coefficient of -1.84 (*p*< 0.001). However, T had no significant effect on SA and SPAD, with path coefficients of -0.17 (*p* > 0.05) and -0.48 (*p* > 0.05), respectively. Additionally, a significant positive correlation was observed between SOD and SPAD, with a path coefficient of 0.89 (*p*< 0.001), suggesting that increased SOD activity significantly co-occurred with higher SPAD values. Furthermore, both SOD and SPAD had significant direct positive effects on Pn, with path coefficients of 0.40 (*p*< 0.001) and 0.21 (*p*< 0.001), respectively. The R² analysis indicated that the model explained 98.1% (R^2^ = 0.981) of the variance in Pn.

**Figure 9:**
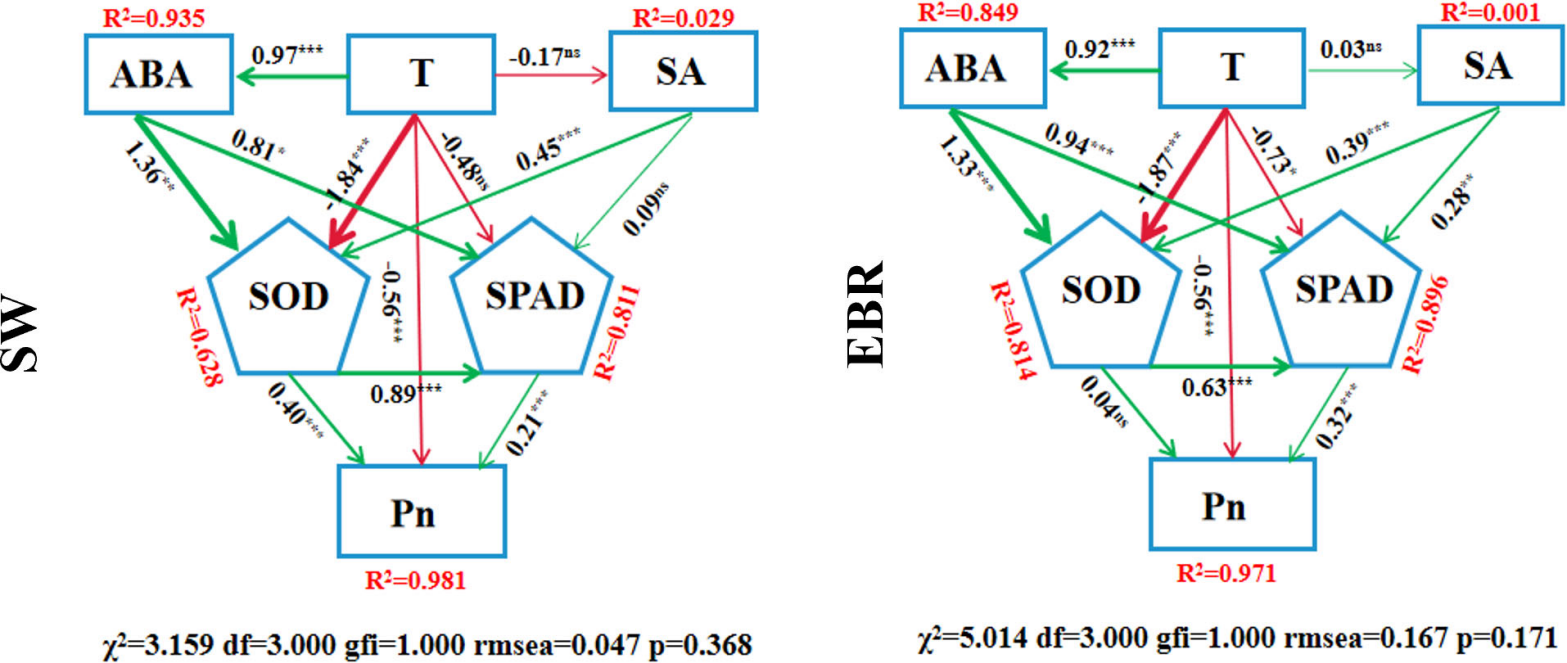
Structural equation model. T represents leaf temperature; ABA represents abscisic acid; SA represents salicylic acid; SOD represents superoxide dismutase; Pn represents net photosynthetic rate; X^2^ represents chi-square statistic; df represents degrees of freedom of the model; gfi represents goodness of fit index; rmsea represents root mean square error of approximation; p represents *P*-value of the chi-square test. * represents *p*< 0.05, ** represents *p*< 0.01, *** represents *p*< 0.001, and ns represents *p* > 0.05. SW represent spraying water; EBR represent spraying 24-epibrassinolide.

Under EBR treatment, the model showed a reasonable fit (χ^2^ = 5.014, df = 3.000, GFI = 1.000, RMSEA = 0.167, p = 0.171). The positive relationship between T and ABA remained significant under this condition, with a path coefficient of 0.92 (*p*< 0.001), indicating that an increase in T still significantly enhanced ABA accumulation. However, the effect of T on SA was insignificant, with a path coefficient of 0.03 (*p* > 0.05). A significant negative relationship between T and SPAD also emerged, with a path coefficient of -0.73 (*p*< 0.05), while the negative effect of T on SOD remained significant, with a path coefficient of -1.87 (*p*< 0.001). The positive correlation between SOD and SPAD was still significant, with a path coefficient of 0.63 (*p*< 0.001), showing that SOD activity was positively associated with SPAD. This result was consistent with the SW treatment. However, unlike the SW treatment, SOD had no significant direct effect on Pn (path coefficient 0.04, *p* > 0.05), whereas the effect of SPAD on Pn was enhanced under EBR treatment, with a path coefficient of 0.32 (*p*< 0.001). R^2^ analysis revealed that the model explained 97.1% (R^2^ = 0.971) of the variance in Pn under EBR treatment. Overall, both under SW and EBR treatments, T and ABA showed a significant positive regulatory relationship, suggesting that T plays a crucial role in regulating ABA synthesis or accumulation. Compared to SW treatment, EBR positively regulated the effect of the SA pathway on SPAD values and the influence of SPAD on Pn, enhancing wheat’s ability to regulate hormones in response to DH stress and thereby improving its capacity to cope with such conditions. Additionally, EBR reduced the impact of antioxidant enzyme pathways, such as SOD, on the net photosynthetic rate, alleviating the damage caused by DH and decreasing wheat’s reliance on antioxidant enzymes to maintain Pn (**Figure 9**).

## 4 Discussions

### 4.1 Effects of EBR on the homeostasis of endogenous hormones in wheat flag leaves subjected to dry–hot wind stress

The accumulation of ABA and JA in wheat flag leaves decreased, while the accumulation of SA increased under EBR treatment (**Figure 2**).

Under stress conditions such as high temperature, drought (Kong et al., 2021), and high salinity (Kaur and Asthir, 2020), ABA rapidly accumulates in wheat, activating signaling pathways that enhance stress adaptation and growth activity, thereby promoting plant growth under stress. Both JA (Pedranzani et al., 2003; De Ollas et al., 2013; Yan et al., 2015; Ali and Baek, 2020) and SA (Fayez and Bazaid, 2014; Dong et al., 2014; Yadava et al., 2015; Wang et al., 2018; La et al., 2019; Fan et al., 2022) play pivotal roles in mediating responses to abiotic stresses, including salinity, drought, waterlogging, heavy metals, and temperature variations. Prior research has established an antagonistic relationship between ABA and BRs during plant growth and development. EBR treatment can downregulate the expression of ABA pathway-regulated transcription factors (BrABI1, BrABI2, BrABI5) in fresh daylily flower buds, leading to a significant reduction in endogenous ABA content (Yao et al., 2017). In Arabidopsis, EBR can stimulate the SA-sensing pathway in response to stress (Divi et al., 2010). In cotton, the biotic -stress-induced transcription factor GhTINY2 orchestrates a balance between SA-mediated immune responses and BR-regulated growth and defense mechanisms (Xiao et al., 2021). The intricate interplay between the BRs and JA pathways is exemplified by their inhibitory effects on root elongation in both wild-type Arabidopsis and the *psc1coi1* mutant, mediated by JA (Ren et al., 2009). While in bok choy, EBR delays leaf senescence by antagonizing JA and ABA (Zhu et al., 2023). In wheat, consistent with previous studies in rice and Arabidopsis (Liao et al., 2020; Li et al., 2021), EBR treatment resulted in a reduction in the levels of ABA and JA in the leaves (**Figures 2A, B**). The decrease in ABA content led to an increase in gs, which subsequently enhanced Tr, reduced spike leaf temperature, and contributed to an increase in antioxidant enzyme activities, ultimately improving Pn. Moreover, the elevation in SA levels indicates that wheat’s stress resistance was significantly enhanced under EBR treatment. This suggests that EBR can enhance wheat resistance to DH by modulating the hormonal balance.

### 4.2 Regulatory effects of EBR on photosynthesis and canopy temperature in wheat subjected to dry–hot wind stress

BRs are capable of downregulating ABA biosynthesis genes, leading to a reduced ABA content in plants, and they can directly influence stomata, regulating their aperture. Previous studies have demonstrated that low-concentration applications of BRs on tomatoes promote stomatal opening (Xia et al., 2014), and EBR has been specifically shown to increase gs in melon leaves under high-temperature stress (Zhang et al., 2013). In the current experiment, the EBR concentration used was 0.1 mg·L^-1^, which falls within the range of low concentrations known to directly regulate stomatal opening. Under DH stress conditions, the Tr values of flag leaves were significantly reduced in both wheat cultivars. However, EBR effectively alleviated the negative impacts of DH on Tr (**Figure 3**). Furthermore, DH treatment markedly increased spike and leaf temperatures in wheat, whereas EBR treatment significantly mitigated these temperature increases compared to SW (**Figure 4**). The enhancement of Tr in wheat by EBR can be attributed to its capacity to increase gs. Tr represents the amount of water transpired per unit leaf area over a specified time interval, and the transpired water acts as a heat dissipater, thereby enabling EBR to lower spike and leaf temperatures in wheat and mitigate heat stress. Additionally, it was previously established that EBR can elevate gs, proline content, and relative water content in mustard plants subjected to drought stress (Fariduddin et al., 2009). These findings are consistent with the current experimental results, which show that EBR increases the flag leaf Tr in wheat, thereby reducing heat stress on intracellular proteins and enzymes. Collectively, these results suggest that EBR plays a crucial role in mitigating the detrimental effects of DH stress on wheat physiology by modulating stomatal function and enhancing water relations.

However, it is worth noting a critical issue in this experiment—namely, the impact of the covering film on photosynthetically active radiation (PAR) within the greenhouse. This factor should be addressed because the light transmittance of the covering film led to a reduction in PAR, which subsequently altered the original physiological processes of wheat under DH conditions. As a result, the diminished photosynthetic capacity of wheat under DH stress can be partly attributed to the reduction in PAR. Nevertheless, previous studies suggest that the use of polyethylene films in drought tolerance research is a well-established and viable approach (Al-Madani et al., 2024). Moreover, the data in this study indicate that EBR treatment significantly improved both hormonal regulation (**Figure 2**) and photosynthetic capacity (**Figures 3**, **6**, **7**) of wheat under DH conditions compared to the SW treatment. Although the PAR inside the greenhouse was indeed reduced compared to that under FC conditions, this does not impede the investigation of EBR’s effects under DH stress.

### 4.3 Regulatory effects of EBR on the antioxidant system, SPAD values, and chlorophyll fluorescence in wheat flag leaves under dry–hot wind stress

The activities of antioxidant enzymes, including SOD, POD, and CAT, in wheat flag leaves were notably augmented under EBR treatment (**Figure 5**).

Abiotic stress often leads to ROS accumulation in wheat, inducing oxidative stress and compromising plant growth and development (Ru et al., 2023). Wheat maintains ROS homeostasis through its endogenous antioxidant enzyme system, particularly SOD, CAT, and POD (Miller et al., 2010). Prior studies have shown that, in Pinellia ternata EBR upregulates the expression of *FeSOD*, *POD*, and *CAT* genes, enhancing proline synthesis (mediated by P5CS1) (Guo et al., 2022), increasing gs and Tr (**Figure 3**). In the early stages of stress, SOD (**Figure 5A**), POD (**Figure 5B**), and CAT (**Figure 5C**) activities increased significantly to counteract ROS accumulation in the wheat cultivars under DH conditions. EBR further elevated these activities, consistent with previous findings (Khan et al., 2022).

Chlorophyll fluorescence parameters, particularly Fv/Fm and PIabs, provide insights into the intricate interplay between plant photosynthesis and environmental stresses (Kalaji et al., 2016). Fv/Fm serves as a proxy for the primary photochemical conversion efficiency of PSII, whereas PIabs values more intimately reflect the consequences of external stress on the photosynthetic machinery (Van Heerden et al., 2004). In the context of leaf senescence, chlorophyll degradation emerges as a pivotal metabolic event, with a decline in total SPAD value (Relative chlorophyll content) serving as a reliable indicator of this process (Hörtensteiner, 2006).

Wheat plants possess the capacity to maintain SPAD value during the initial phases of stress exposure through self-regulation (**Figure 6**). However, as stress intensity escalates beyond the plant’s tolerance threshold, chlorophyll degradation commences, accelerating leaf senescence. Notably, EBR significantly elevated the SPAD, Fv/Fm, and PIabs values of flag leaves in both wheat cultivars (**Figures 6**, **7**). This enhancement can be attributed to the following two primary mechanisms: firstly, spike and leaf temperatures are mitigated (**Figure 4**), alleviating heat stress and consequently slowing chlorophyll degradation (**Figure 6**). Secondly, the increase in antioxidant enzyme activities (**Figure 5**) has effectively inhibited the accumulation of ROS. Furthermore, EBR treatment upregulates the expression of chlorophyll synthesis genes, contributing to an increase in the relative chlorophyll content (Peng et al., 2020). Additionally, EBR promotes thermal dissipation in plants, safeguarding the PSII reaction center and electron transport chain (Hu et al., 2013; Lima and Lobato, 2017), thereby enhancing both the “quantity” and “quality” of chlorophyll in a dual manner.

### 4.4 Regulatory effects of EBR on dry matter transport and yield in wheat subjected to dry–hot wind stress

DH stress affects wheat yield by reducing the grain weight of the two cultivars, with no notable impact on the number of spikes or grains per spike (**Table 5**).

Under DH conditions, the SPAD of the wheat plants decreased, leading to an insufficiency of antenna pigment molecules essential for light energy absorption. Consequently, the Fv/Fm and PIabs values of the sheltered wheat cultivars were also lower, indicating a weakened capacity of PSII to capture and transfer electrons. This diminished electron transfer resulted in insufficient chemical energy for the carbon fixation reaction, ultimately causing a significant reduction in the accumulation of total dry matter and grain weight compared to FC-grown wheat. In contrast, treatment with EBR effectively mitigated the adverse effects of DH stress in both wheat cultivars. Specifically, the variation in the ratio between grain yield and dry matter was low. This stability in the ratio indicates that the effects of the treatment on dry matter were effectively transmitted to the yield. The effect of EBR is not limited to influencing a single physiological parameter but enhanced the translocation of dry matter from various vegetative organs to the grains (**Tables 2**–**4**), thereby increasing grain weight. This finding aligns with previous studies reporting that treatment with 0.1 mg·L^-1^ EBR resulted in grain yield increments of 18% and 20% upon cessation of irrigation during the flowering and grain filling stages of wheat (Dehghan et al., 2020). Importantly, EBR treatment had no significant effects on the number of spikes or grains per spike (**Table 5**). Therefore, the yield-enhancing effect of EBR under DH stress was achieved primarily through the optimization of grain filling processes rather than by modulating spike or grain number.

### 4.5 Differential responses of dry–hot wind-sensitive and resistant wheat cultivars to EBR treatment

EBR treatment alleviated dry–hot wind stress in both wheat cultivars with different resistance levels; however, its regulatory effects differed significantly between JN17 and LX77. Specifically, under EBR treatment, physiological parameters (such as photosynthetic parameters and SPAD values) and antioxidant enzyme activities (SOD, POD, and CAT) exhibited a significantly greater increase in the sensitive cultivar JN17 than in the resistant cultivar LX77 (**Figures 3**, **5**–**7**). This suggests that EBR may have a more pronounced relative effect in enhancing the tolerance of dry–hot wind-sensitive cultivars. This phenomenon may be attributed to the lower intrinsic resistance of sensitive cultivars to dry–hot wind stress, which allows their physiological and metabolic pathways to be more effectively activated and optimized in response to EBR treatment. In contrast, LX77 exhibited superior stomatal regulation, antioxidant systems, and photosynthetic capacity compared to the sensitive cultivar JN17. Although EBR treatment still exerted positive effects on LX77, such as improving PSII activity, enhancing photosynthesis, and increasing antioxidant enzyme activities, the overall improvement was relatively smaller. This indicates that EBR may have a “response threshold” or “effect saturation” in highly resistant wheat cultivars. That is, while EBR can enhance stress tolerance to a certain extent, its ability to further improve resistance in cultivars that already possess strong tolerance mechanisms may be limited. Furthermore, the differential effects of EBR treatment in the two cultivars may also be related to differences in endogenous hormone regulation mechanisms. Different wheat genotypes may exhibit varying sensitivities to EBR-induced hormone signaling pathways, potentially leading to a stronger hormonal response and physiological improvement in the sensitive cultivar after EBR treatment, whereas the response amplitude in the resistant cultivar remains relatively smaller. The model suggests a common regulatory pathway for EBR-mediated stress tolerance (**Figure 9**); however, the response may vary across wheat genotypes. In this study, the JN17 exhibited a more pronounced improvement compared to the LX77, indicating that genotype-specific differences in stress perception and physiological regulation may influence EBR effectiveness. Future studies incorporating a wider range of wheat genotypes and molecular-level analyses are needed to refine the model and assess its broader applicability.

### 4.6 Limitations of this study and the large-scale application and diffusion of EBR

While this study provides insights into the effects of EBR on wheat under DH, certain limitations must be acknowledged. First, the study did not evaluate transcription factors or ABA/JA/SA-responsive genes, which limits a deeper understanding of how EBR regulates molecular mechanisms involving endogenous hormones such as ABA, JA, and SA. Furthermore, the use of covering films in the experiments may have disrupted PAR, potentially affecting the plants’ photosynthetic capacity. This study was also limited to only two wheat cultivars, which may constrain the generalizability of the findings to other cultivars. In addition, wind speed was not recorded during the field trials, as natural wind fluctuations were inherently variable and typically below the threshold required to induce significant heat stress. Nevertheless, the consistency of cultivation methods and control of key environmental parameters ensured the reliability and comparability of data across the treatment groups.

EBR is a promising plant growth regulator with significant potential to enhance crop stress tolerance, improve yield, and promote growth in diverse agricultural systems. However, several technical and economic challenges remain for its large-scale application. These include issues related to cost-effectiveness, environmental adaptability, and differences in the mode of action between crop species. Additionally, EBR performance must be rigorously evaluated under varying agricultural conditions to ensure its sustainability and broad applicability. Considering the practical agricultural environment, the field trial design in this study did not control for temperature, humidity, or wind speed, effectively simulating real-world farming conditions. This approach captured the complex interactions between external environmental variables and crop performance, thereby providing a more reliable reference point for the practical application of EBR in agriculture. Nonetheless, future research should emphasize the monitoring and control of environmental variables, particularly through comparative experiments conducted in controlled or temperature-regulated environments, to more accurately dissect the underlying mechanisms of EBR action. Investigating EBR’s efficacy under diverse environmental conditions will further substantiate its adaptability and stability in dynamic agricultural settings.

In conclusion, while EBR hold great promise in improving crop resilience and productivity, overcoming the current technical and economic barriers is essential for their widespread adoption. Future studies should prioritize optimizing EBR application strategies, assessing its performance across different crops and environmental conditions, and addressing cost-related challenges to ensure its sustainable and broad application in modern agriculture.

## 5 Conclusions

**Figure 10:**
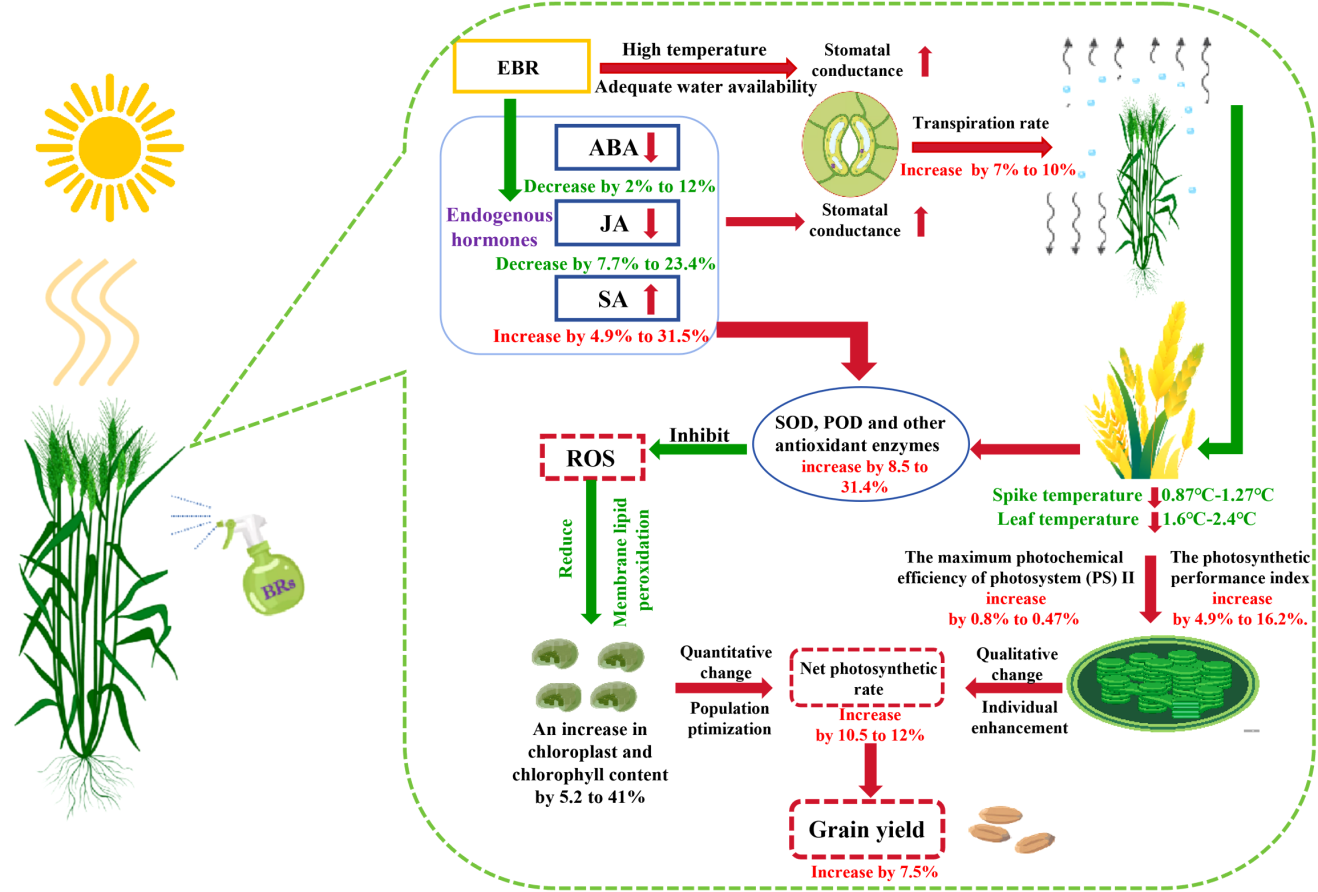
Pattern diagram. EBR, 24-epibrassinolide; ABA, abscisic acid; JA, jasmonic acid; SA, salicylic acid; SOD, superoxide dismutase; POD, peroxidase; ROS, reactive oxygen species.

EBR has shown significant effects in enhancing wheat resistance to DH stress; this mechanism lies in its ability to effectively reduce the ABA content in wheat flag leaves, thereby promoting stomatal opening, increasing the Tr, and ultimately achieving a significant reduction in the spike and leaf temperatures. This process effectively alleviates the heat stress caused by dry–hot wind, stabilizes the antioxidant enzyme activity in wheat, thereby significantly enhancing wheat’s resistance to dry–hot wind. Further studies have found that EBR can increase the relative chlorophyll content in wheat flag leaves, which directly enhances their Fv/Fm and PIabs values. During the grain filling period of wheat, this effect significantly enhances the dry matter transfer capacity under dry–hot wind stress, providing strong support for wheat yield and quality. Notably, EBR also enhances wheat’s hormonal regulation abilities by increasing the path coefficient of SA to SPAD and SPAD to Pn values. This enhanced regulatory ability effectively maintains the Pn of wheat, thereby significantly mitigating the adverse effects of dry–hot wind on wheat growth and development. To present a deeper understanding of the mitigating effects of EBR on wheat under dry–hot wind stress, the schematic diagram presented in **Figure 10** was constructed in this study.

## Data Availability

The original contributions presented in the study are included in the article/supplementary material. Further inquiries can be directed to the corresponding authors.

## References

[B1] Akter N. Rafiqul Islam M. (2017). Heat stress effects and management in wheat. A review. Agron. Sustain. Dev. 37, 37. doi: 10.1007/s13593-017-0443-9

[B2] Alam P. Albalawi T. H. Altalayan F. H. Bakht M. A. Ahanger M. A. Raja V.. (2019). 24-epibrassinolide (EBR) confers tolerance against naCl stress in soybean plants by up-regulating antioxidant system, ascorbate-glutathione cycle, and glyoxalase system. Biomolecules 9, 640. doi: 10.3390/biom9110640 31652728 PMC6920941

[B3] Ali M. Baek K.-H. (2020). Jasmonic acid signaling pathway in response to abiotic stresses in plants. IJMS 21, 621. doi: 10.3390/ijms21020621 31963549 PMC7013817

[B4] Al-Madani A. A. Al-Helal I. M. Alsadon A. A. (2024). Assessing the effectiveness of reflective and diffusive polyethylene films as greenhouse covers in arid environments. Agronomy 14, 1082. doi: 10.3390/agronomy14051082

[B5] Anjum S. A. Tanveer M. Ashraf U. Hussain S. Shahzad B. Khan I.. (2016). Effect of progressive drought stress on growth, leaf gas exchange, and antioxidant production in two maize cultivars. Environ. Sci. pollut. Res. 23, 17132–17141. doi: 10.1007/s11356-016-6894-8 27215981

[B6] Ashraf M. Ali Q. (2008). Relative membrane permeability and activities of some antioxidant enzymes as the key determinants of salt tolerance in canola (*Brassica napus* L.). Environ. Exp. Bot. 63, 266–273. doi: 10.1016/j.envexpbot.2007.11.008

[B7] Chaves M. M. Flexas J. Pinheiro C. (2009). Photosynthesis under drought and salt stress: regulation mechanisms from whole plant to cell. Ann. Bot. 103, 551–560. doi: 10.1093/aob/mcn125 18662937 PMC2707345

[B8] Choudhary S. P. Yu J.-Q. Yamaguchi-Shinozaki K. Shinozaki K. Tran L.-S. P. (2012). Benefits of brassinosteroid crosstalk. Trends Plant Sci. 17, 594–605. doi: 10.1016/j.tplants.2012.05.012 22738940

[B9] Clouse S. D. (1996). Molecular genetic studies confirm the role of brassinosteroids in plant growth and development. Plant J. 10, 1–8. doi: 10.1046/j.1365-313X.1996.10010001.x 8758975

[B10] Czarnocka W. Karpiński S. (2018). Friend or foe? Reactive oxygen species production, scavenging and signaling in plant response to environmental stresses. Free Radical Biol. Med. 122, 4–20. doi: 10.1016/j.freeradbiomed.2018.01.011 29331649

[B11] Dave A. Hernández M. L. He Z. Andriotis V. M. E. Vaistij F. E. Larson T. R.. (2011). 12-oxo-phytodienoic acid accumulation during seed development represses seed germination in *arabidopsis* . Plant Cell 23, 583–599. doi: 10.1105/tpc.110.081489 21335376 PMC3077774

[B12] Dehghan M. Balouchi H. Yadavi A. Zare E. (2020). Improve wheat (*Triticum aestivum*) performance by brassinolide application under different irrigation regimes. South Afr. J. Bot. 130, 259–267. doi: 10.1016/j.sajb.2020.01.013

[B13] De Ollas C. Hernando B. Arbona V. Gómez-Cadenas A. (2013). Jasmonic acid transient accumulation is needed for abscisic acid increase in citrus roots under drought stress conditions. Physiologia Plantarum 147, 296–306. doi: 10.1111/j.1399-3054.2012.01659 22671923

[B14] Divi U. K. Rahman T. Krishna P. (2010). Brassinosteroid-mediated stress tolerance in Arabidopsis shows interactions with abscisic acid, ethylene and salicylic acid pathways. BMC Plant Biol. 10, 151. doi: 10.1186/1471-2229-10-151 20642851 PMC3095295

[B15] Dong C.-J. Li L. Shang Q.-M. Liu X.-Y. Zhang Z.-G. (2014). Endogenous salicylic acid accumulation is required for chilling tolerance in cucumber (*Cucumis sativus* L.) seedlings. Planta 240, 687–700. doi: 10.1007/s00425-014-2115-1 25034826

[B16] Dong H. Liu J. He G. Liu P. Sun J. (2020). Photoexcited phytochrome B interacts with brassinazole resistant 1 to repress brassinosteroid signaling in *Arabidopsis* . JIPB 62, 652–667. doi: 10.1111/jipb.12822 31081597

[B17] Engelberth J. Schmelz E. A. Alborn H. T. Cardoza Y. J. Huang J. Tumlinson J. H. (2003). Simultaneous quantification of jasmonic acid and salicylic acid in plants by vapor-phase extraction and gas chromatography-chemical ionization-mass spectrometryq. Analytical Biochemistry 31 (2), 242–250. doi: 10.1016/S0003-2697(02)00466-9 12531212

[B18] Fan Y. Lv Z. Li Y. Qin B. Song Q. Ma L.. (2022). Salicylic acid reduces wheat yield loss caused by high temperature stress by enhancing the photosynthetic performance of the flag leaves. Agronomy 12, 1386. doi: 10.3390/agronomy12061386

[B19] Fariduddin Q. Khanam S. Hasan S. A. Ali B. Hayat S. Ahmad A. (2009). Effect of 28-homobrassinolide on the drought stress-induced changes in photosynthesis and antioxidant system of Brassica juncea L. Acta Physiol. Plant 31, 889–897. doi: 10.1007/s11738-009-0302-7

[B20] Fayez K. A. Bazaid S. A. (2014). Improving drought and salinity tolerance in barley by application of salicylic acid and potassium nitrate. J. Saudi Soc. Agric. Sci. 13, 45–55. doi: 10.1016/j.jssas.2013.01.001

[B21] Guo H. Chen H. Hong C. Jiang D. Zheng B. (2017). Exogenous Malic acid alleviates cadmium toxicity in Miscanthus sacchariflorus through enhancing photosynthetic capacity and restraining ROS accumulation. Ecotoxicol. Environ. Saf. 141, 119–128. doi: 10.1016/j.ecoenv.2017.03.018 28324818

[B22] Guo C. Chen Y. Wang M. Du Y. Wu D. Chu J.. (2022). Exogenous brassinolide improves the antioxidant capacity of Pinellia ternata by enhancing the enzymatic and nonenzymatic defense systems under non-stress conditions. Front. Plant Sci. 13. doi: 10.3389/fpls.2022.917301 PMC9358693 35958199

[B23] Ha Y. M. Shang Y. Yang D. Nam K. H. (2018). Brassinosteroid reduces ABA accumulation leading to the inhibition of ABA-induced stomatal closure. Biochem. Biophys. Res. Commun. 504, 143–148. doi: 10.1016/j.bbrc.2018.08.146 30170727

[B24] Hörtensteiner S. (2006). Chlorophyll degradation during senescence. Annu. Rev. Plant Biol. 57, 55–77. doi: 10.1146/annurev.arplant.57.032905.105212 16669755

[B25] Hu J. Huang J. Xu H. Wang Y. Li C. Wen P.. (2020). Rice stripe virus suppresses jasmonic acid-mediated resistance by hijacking brassinosteroid signaling pathway in rice. PloS Pathog. 16, e1008801. doi: 10.1371/journal.ppat.1008801 32866183 PMC7485985

[B26] Hu W. Yan X. Xiao Y. Zeng J. Qi H. Ogweno J. O. (2013). 24-Epibrassinosteroid alleviate drought-induced inhibition of photosynthesis in Capsicum annuum. Scientia Hortic. 150, 232–237. doi: 10.1016/j.scienta.2012.11.012

[B27] Hussain S. Nanda S. Ashraf M. Siddiqui A. Masood S. Khaskheli M.. (2023). Interplay impact of exogenous application of abscisic acid (ABA) and brassinosteroids (BRs) in rice growth, physiology, and resistance under sodium chloride stress. Life 13, 498. doi: 10.3390/life13020498 36836855 PMC9965451

[B28] Kalaji H. M. Jajoo A. Oukarroum A. Brestic M. Zivcak M. Samborska I. A.. (2016). Chlorophyll a fluorescence as a tool to monitor physiological status of plants under abiotic stress conditions. Acta Physiol. Plant 38, 102. doi: 10.1007/s11738-016-2113-y

[B29] Kang Y. H. Breda A. Hardtke C. S. (2017). Brassinosteroid signaling directs formative cell divisions and protophloem differentiation in *Arabidopsis* root meristems. Development 144, 272–280. doi: 10.1242/dev.145623 28096215 PMC5394764

[B30] Karlidag H. Yildirim E. Turan M. (2011). Role of 24-epibrassinolide in mitigating the adverse effects of salt stress on stomatal conductance, membrane permeability, and leaf water content, ionic composition in salt stressed strawberry (Fragaria× ananassa). Scientia Hortic. 130, 133–140. doi: 10.1016/j.scienta.2011.06.025

[B31] Kaur G. Asthir B. (2020). Impact of exogenously applied ABA on proline metabolism conferring drought and salinity stress tolerance in wheat genotypes. Cereal Res. Commun. 48, 309–315. doi: 10.1007/s42976-020-00041-0

[B32] Kaur P. Bali S. Sharma A. Kohli S. K. Vig A. P. Bhardwaj R.. (2019). Cd induced generation of free radical species in Brassica juncea is regulated by supplementation of earthworms in the drilosphere. Sci. Total Environ. 655, 663–675. doi: 10.1016/j.scitotenv.2018.11.096 30476847

[B33] Khan T. A. Ahmad A. Saeed T. Yusuf M. Faisal M. Alatar A. A. (2024). Investigating the influence of selenium and epibrassinolide on antioxidant activity, proline accumulation, and protein expression profiles in wheat plants experiencing heat and drought stress. Front. Plant Sci. 15. doi: 10.3389/fpls.2024.1441483 PMC11534860 39502922

[B34] Khan R. Ma X. Hussain Q. Asim M. Iqbal A. Ren X.. (2022). Application of 2,4-epibrassinolide improves drought tolerance in tobacco through physiological and biochemical mechanisms. Biology 11, 1192. doi: 10.3390/biology11081192 36009819 PMC9405153

[B35] Kim Y.-W. Youn J.-H. Roh J. Kim J.-M. Kim S.-K. Kim T.-W. (2022). Brassinosteroids enhance salicylic acid-mediated immune responses by inhibiting BIN2 phosphorylation of clade I TGA transcription factors in Arabidopsis. Mol. Plant 15, 991–1007. doi: 10.1016/j.molp.2022.05.002 35524409

[B36] Kohli S. K. Handa N. Sharma A. Kumar V. Kaur P. Bhardwaj R. (2017). Synergistic effect of 24-epibrassinolide and salicylic acid on photosynthetic efficiency and gene expression in Brassica juncea L. under Pb stress. Turk J. Biol. 41, 943–953. doi: 10.3906/biy-1707-15 30814859 PMC6353293

[B37] Kong H. Zhang Z. Qin J. Akram N. A. (2021). Synergistic effects of abscisic acid (ABA) and drought stress on the physiological responses of winter wheat (*Triticum aestivum* L.). PAK.J.BOT 53 (5), 1545–1551. doi: doi: 10.30848/PJB2021-5(11)

[B38] La V. H. Lee B.-R. Islam M. Park S.-H. Jung H. Bae D.-W.. (2019). Characterization of salicylic acid-mediated modulation of the drought stress responses: Reactive oxygen species, proline, and redox state in Brassica napus. Environ. Exp. Bot. 157, 1–10. doi: 10.1016/j.envexpbot.2018.09.013

[B39] Lawlor D. W. (2009). Musings about the effects of environment on photosynthesis. Ann. Bot. 103, 543–549. doi: 10.1093/aob/mcn256 19205084 PMC2707351

[B40] Li M. Wei Q. Zhu Y. Li J. Ullah N. Song Y. (2023). 24-Epicastasterone and KH_2_ PO_4_ protect grain production of wheat crops from terminal heat impacts by modulating leaf physiology. Arch. Agron. Soil Sci. 69, 2006–2019. doi: 10.1080/03650340.2022.2130265

[B41] Li Q. Xu F. Chen Z. Teng Z. Sun K. Li X.. (2021). Synergistic interplay of ABA and BR signal in regulating plant growth and adaptation. Nat. Plants 7, 1108–1118. doi: 10.1038/s41477-021-00959-1 34226689

[B42] Liao K. Peng Y.-J. Yuan L.-B. Dai Y.-S. Chen Q.-F. Yu L.-J.. (2020). Brassinosteroids antagonize jasmonate-activated plant defense responses through BRI1-EMS-SUPPRESSOR1 (BES1). Plant Physiol. 182, 1066–1082. doi: 10.1104/pp.19.01220 31776183 PMC6997682

[B43] Liaqat S. Umar S. Saffeullah P. Iqbal N. Siddiqi T. O. khan M. I. R (2020). Protective effect of 24-epibrassinolide on barley plants growing under combined stress of salinity and potassium deficiency. J. Plant Growth Regul. 39, 1543–1558. doi: 10.1007/s00344-020-10163-8

[B44] Lima J. V. Lobato A. K. S. (2017). Brassinosteroids improve photosystem II efficiency, gas exchange, antioxidant enzymes and growth of cowpea plants exposed to water deficit. Physiol. Mol. Biol. Plants 23, 59–72. doi: 10.1007/s12298-016-0410-y 28250584 PMC5313414

[B45] Liu Y. Zhao Z. Si J. Di C. Han J. An L. (2009). Brassinosteroids alleviate chilling-induced oxidative damage by enhancing antioxidant defense system in suspension cultured cells of Chorispora bungeana. Plant Growth Regul. 59, 207–214. doi: 10.1007/s10725-009-9405-9

[B46] Mazorra L. M. Nunez M. Hechavarria M. Coll F. Sanchez-blanco M. J. (2002). Influence of brassinosteroids on antioxidant enzymes activity in tomato under different temperatures. Biol. Plant 45, 593–596. doi: 10.1023/A:1022390917656

[B47] Miller G. A. D. Suzuki N. Ciftci-Yilmaz S. Mittler R. (2010). Reactive oxygen species homeostasis and signalling during drought and salinity stresses. Plant Cell Environ. 33, 453–467. doi: 10.1111/j.1365-3040.2009.02041 19712065

[B48] Nolan T. M. Brennan B. Yang M. Chen J. Zhang M. Li Z.. (2017). Selective autophagy of BES1 mediated by DSK2 balances plant growth and survival. Dev. Cell 41, 33–46.e7. doi: 10.1016/j.devcel.2017.03.013 28399398 PMC5720862

[B49] Pedranzani H. Racagni G. Alemano S. Miersch O. Ramírez I. Peña-Cortés H.. (2003). No title found. Plant Growth Regul. 41, 149–158. doi: 10.1023/A:1027311319940

[B50] Peng R. Sun W. Jin X. Yu L. Chen C. Yue Z.. (2020). Analysis of 2,4-epibrassinolide created an enhancement tolerance on Cd toxicity in Solanum nigrum L. Environ. Sci. pollut. Res. 27, 16784–16797. doi: 10.1007/s11356-020-08228-y 32141006

[B51] Peres A. L. G. L. Soares J. S. Tavares R. G. Righetto G. Zullo M. A. T. Mandava N. B.. (2019). Brassinosteroids, the sixth class of phytohormones: A molecular view from the discovery to hormonal interactions in plant development and stress adaptation. IJMS 20, 331. doi: 10.3390/ijms20020331 30650539 PMC6359644

[B52] Ren C. Han C. Peng W. Huang Y. Peng Z. Xiong X.. (2009). A leaky mutation in *DWARF4* reveals an antagonistic role of brassinosteroid in the inhibition of root growth by jasmonate in arabidopsis. Plant Physiol. 151, 1412–1420. doi: 10.1104/pp.109.140202 19741050 PMC2773060

[B53] Retzer K. Akhmanova M. Konstantinova N. Malínská K. Leitner J. Petrášek J.. (2019). Brassinosteroid signaling delimits root gravitropism via sorting of the Arabidopsis PIN2 auxin transporter. Nat. Commun. 10, 5516. doi: 10.1038/s41467-019-13543-1 31797871 PMC6892858

[B54] Rosseel Y. (2012). lavaan : an *R* package for structural equation modeling. J. Stat. Soft. 48 (2). doi: 10.18637/jss.v048.i02

[B55] Ru C. Hu X. T. Chen D. Y. Wang W. Zhen J. B. (2023). Photosynthetic, antioxidant activities, and osmoregulatory responses in winter wheat differ during the stress and recovery periods under heat, drought, and combined stress. Plant Sci., 11557–111557. doi: 10.1016/j.plantsci.2022.111557 36481364

[B56] Ruley A. T. Sharma N. C. Sahi S. V. (2004). Antioxidant defense in a lead accumulating plant, Sesbania drummondii. Plant Physiol. Biochem. 42, 899–906. doi: 10.1016/j.plaphy.2004.12.001 15694284

[B57] Sade N. Gebretsadik M. Seligmann R. Schwartz A. Wallach R. Moshelion M. (2009). The role of tobacco aquaporin1 in improving water use efficiency, hydraulic conductivity, and yield production under salt stress. Plant Physiol. 152, 245–254. doi: 10.1104/pp.109.145854 19939947 PMC2799360

[B58] Sharma A. Shahzad B. Kumar V. Kohli S. K. Sidhu G. P. S. Bali A. S.. (2019). Phytohormones regulate accumulation of osmolytes under abiotic stress. Biomolecules 9, 285. doi: 10.3390/biom9070285 31319576 PMC6680914

[B59] Sharma A. Thakur S. Kumar V. Kesavan A. K. Thukral A. K. Bhardwaj R. (2017). 24-epibrassinolide stimulates imidacloprid detoxification by modulating the gene expression of Brassica juncea L. BMC Plant Biol. 17, 56. doi: 10.1186/s12870-017-1003-9 28245791 PMC5477812

[B60] Siddiqui H. Hayat S. Bajguz A. (2018). Regulation of photosynthesis by brassinosteroids in plants. Acta Physiol. Plant 40, 59. doi: 10.1007/s11738-018-2639-2

[B61] Šimonovičová M. Tamás L. Huttová J. Mistrík I. (2004). Effect of aluminium on oxidative stress related enzymes activities in barley roots. Biol. Plant 48, 261–266. doi: 10.1023/B:BIOP.0000033454.95515.8a

[B62] Soares C. De Sousa A. Pinto A. Azenha M. Teixeira J. Azevedo R. A.. (2016). Effect of 24-epibrassinolide on ROS content, antioxidant system, lipid peroxidation and Ni uptake in Solanum nigrum L. under Ni stress. Environ. Exp. Bot. 122, 115–125. doi: 10.1016/j.envexpbot.2015.09.010

[B63] Tanveer M. Shahzad B. Sharma A. Biju S. Bhardwaj R. (2018). 24-Epibrassinolide; an active brassinolide and its role in salt stress tolerance in plants: A review. Plant Physiol. Biochem. 130, 69–79. doi: 10.1016/j.plaphy.2018.06.035 29966934

[B64] Unterholzner S. J. Rozhon W. Papacek M. Ciomas J. Lange T. Kugler K. G.. (2015). Brassinosteroids are master regulators of gibberellin biosynthesis in arabidopsis. Plant Cell 27, 2261–2272. doi: 10.1105/tpc.15.00433 26243314 PMC4568508

[B65] Van Heerden P. D. R. Strasser R. J. Krüger G. H. J. (2004). Reduction of dark chilling stress in N2 -fixing soybean by nitrate as indicated by chlorophyll a fluorescence kinetics. Physiologia Plantarum 121, 239–249. doi: 10.1111/j.0031-9317.2004.0312 15153191

[B66] Wang X. Ren Z. B. Xie S. P. Li Z. H. Zhou Y. Y. Duan L. S. (2024). Jasmonate mimic modulates cell elongation by regulating antagonistic bHLH transcription factors via brassinosteroid signaling. Plant Physiol. 195, 2712–2726. doi: 10.1093/plphys/kiae217 38636101

[B67] Wang Y. Wen T. Huang Y. Guan Y. Hu J. (2018). Salicylic acid biosynthesis inhibitors increase chilling injury to maize (*Zea mays* L.) seedlings. Plant Growth Regul. 86, 11–21. doi: 10.1007/s10725-018-0407-3

[B68] WRB. (2015). World Reference Base for Soil Resources 2014: International soil classification systems for naming soils and creating legends for soil maps (Update 2015) (Food Agric. Organ. U. Nations). http://www.fao.org/soils-portal/soil-survey/soil-classification/world-reference-base/en/.

[B69] Wu Z. Liu S. Zhao J. Wang F. Du Y. Zou S.. (2017). Comparative responses to silicon and selenium in relation to antioxidant enzyme system and the glutathione-ascorbate cycle in flowering Chinese cabbage (*Brassica campestris* L. ssp. chinensis var. utilis) under cadmium stress. Environ. Exp. Bot. 133, 1–11. doi: 10.1016/j.envexpbot.2016.09.005

[B70] Xia X. Gao C. Song L. Zhou Y. Shi K. Yu J. (2014). Role of H_2_ O_2_ dynamics in brassinosteroid-induced stomatal closure and opening in * S olanum lycopersicum* . Plant Cell Environ. 37, 2036–2050. doi: 10.1111/pce.12275 24428600

[B71] Xia X. J. Huang Y. Y. Wang L. Huang L. F. Yu Y. L. Zhou Y. H.. (2006). Pesticides-induced depression of photosynthesis was alleviated by 24-epibrassinolide pretreatment in Cucumis sativus L. Pesticide Biochem. Physiol. 86, 42–48. doi: 10.1016/j.pestbp.2006.01.005

[B72] Xiao S. Hu Q. Zhang X. Si H. Liu S. Chen L.. (2021). Orchestration of plant development and defense by indirect crosstalk of salicylic acid and brassinosteorid signaling via transcription factor GhTINY2. J. Exp. Bot. 72, 4721–4743. doi: 10.1093/jxb/erab186 33928361

[B73] Xiong M. Chu L. Li Q. Yu J. Yang Y. Zhou P.. (2021). Brassinosteroid and gibberellin coordinate rice seed germination and embryo growth by regulating glutelin mobilization. Crop J. 9, 1039–1048. doi: 10.1016/j.cj.2020.11.006

[B74] Xiong M. Yu J. Wang J. Gao Q. Huang L. Chen C.. (2022). Brassinosteroids regulate rice seed germination through the BZR1- *RAmy3D* transcriptional module. Plant Physiol. 189, 402–418. doi: 10.1093/plphys/kiac043 35139229 PMC9070845

[B75] Yadava P. Thirunavukkarasu N. Kaur P. Og Shi K. (2015). Salicylsyre lindrer methylviologen-induceret oxidativ stress gennem transkriptionel modulering af antioxidantgener i Zea mays L. Maydica. 60 (3), 1–9. doi: 10.1007/s10725-018-0407-3

[B76] Yan Z. Zhang W. Chen J. Li X. (2015). Methyl jasmonate alleviates cadmium toxicity in Solanum nigrum by regulating metal uptake and antioxidative capacity. Biol. Plant 59, 373–381. doi: 10.1007/s10535-015-0491-4

[B77] Yang J. Miao W. Chen J. (2021). Roles of jasmonates and brassinosteroids in rice responses to high temperature stress – A review. Crop J. 9, 977–985. doi: 10.1016/j.cj.2021.02.007

[B78] Yang C.-J. Zhang C. Lu Y.-N. Jin J.-Q. Wang X.-L. (2011). The mechanisms of brassinosteroids’ Action: from signal transduction to plant development. Mol. Plant 4, 588–600. doi: 10.1093/mp/ssr020 21471332

[B79] Yao Y. Zhao N. Xian T. Tu S. Pan L. Tu K. (2017). Effect of 2,4-epibrassinolide treatment on the postharvest quality and physiological metabolism of fresh daylily flower buds during storage. Scientia Hortic. 226, 110–116. doi: 10.1016/j.scienta.2017.08.039

[B80] Yi X. M. Sun A. Q. Han X. Y. Zhang J. D. Wang Z. L. Wang C. W.. (2015). Identification of heat and drought resistancein major wheat varieties (Lines) promoted in the huang-huai wheat region. J. Triticeae Crops 35 (02), 274–284.

[B81] Zhang M. Zhai Z. Tian X. Duan L. Li Z. (2008). Brassinolide alleviated the adverse effect of water deficits on photosynthesis and the antioxidant of soybean (*Glycine max* L.). Plant Growth Regul. 56, 257–264. doi: 10.1007/s10725-008-9305-4

[B82] Zhang Y. P. Zhu X. H. Ding H. D. Yang S. J. Chen Y. Y. (2013). Foliar application of 24-epibrassinolide alleviates high-temperature-induced inhibition of photosynthesis in seedlings of two melon cultivars. Photosynt 51, 341–349. doi: 10.1007/s11099-013-0031-4

[B83] Zhao N. Zhao M. Tian Y. Wang Y. Han C. Fan M.. (2021). Interaction between BZR1 and EIN3 mediates signalling crosstalk between brassinosteroids and ethylene. New Phytol. 232, 2308–2323. doi: 10.1111/nph.17694 34449890

[B84] Zhong X. Lan R. Su G. Hao L. Xu H. Zhou H.. (2023). Enhancing the salt stress resistance of seeds and seedlings via a brassinolide sustained release agent system. Chem. Biol. Technol. Agric. 10, 140. doi: 10.1186/s40538-023-00510-8

[B85] Zhu J. Liu X. Huang W. An R. Xu X. Li P. (2023). 2,4-Epibrassinolide delays leaf senescence in pak choi (Brassica rapa subsp. chinensis) by regulating its chlorophyll metabolic pathway and endogenous hormones content. Gene 877, 147531. doi: 10.1016/j.gene.2023.147531 37286019

